# Vertebrate Sensory Ganglia: Common and Divergent Features of the Transcriptional Programs Generating Their Functional Specialization

**DOI:** 10.3389/fcell.2020.587699

**Published:** 2020-10-26

**Authors:** Simon Vermeiren, Eric J. Bellefroid, Simon Desiderio

**Affiliations:** ^1^ULB Neuroscience Institute, Université Libre de Bruxelles, Gosselies, Belgium; ^2^Institute for Neurosciences of Montpellier, INSERM U1051, University of Montpellier, Montpellier, France

**Keywords:** cranial sensory ganglia, placodes, neural crest, somatosensory neuron, visceral sensory neuron, nociceptor, transcription factor

## Abstract

Sensory fibers of the peripheral nervous system carry sensation from specific sense structures or use different tissues and organs as receptive fields, and convey this information to the central nervous system. In the head of vertebrates, each cranial sensory ganglia and associated nerves perform specific functions. Sensory ganglia are composed of different types of specialized neurons in which two broad categories can be distinguished, somatosensory neurons relaying all sensations that are felt and visceral sensory neurons sensing the internal milieu and controlling body homeostasis. While in the trunk somatosensory neurons composing the dorsal root ganglia are derived exclusively from neural crest cells, somato- and visceral sensory neurons of cranial sensory ganglia have a dual origin, with contributions from both neural crest and placodes. As most studies on sensory neurogenesis have focused on dorsal root ganglia, our understanding of the molecular mechanisms underlying the embryonic development of the different cranial sensory ganglia remains today rudimentary. However, using single-cell RNA sequencing, recent studies have made significant advances in the characterization of the neuronal diversity of most sensory ganglia. Here we summarize the general anatomy, function and neuronal diversity of cranial sensory ganglia. We then provide an overview of our current knowledge of the transcriptional networks controlling neurogenesis and neuronal diversification in the developing sensory system, focusing on cranial sensory ganglia, highlighting specific aspects of their development and comparing it to that of trunk sensory ganglia.

## Introduction

Sensory perception is of crucial importance for animals to adapt to their environment. Sensory capacities have emerged during animal evolution, as they adopted a more active lifestyle (Patthey et al., 2014). In all species, physical and chemical features of the internal and external environment are detected by specialized sensory cell types (photoreceptors, mechanoreceptors, chemoreceptors, …) often building complex sensory structures (such as the eyes, inner ear and olfactory epithelium). In vertebrates, their appearance together with the parallel development of functional sensory circuits in the PNS and of targets in the CNS have allowed the emergence of reflex circuits and complex survival and social behaviors (Schlosser, 2018).

The PNS comprises all neurons and nerves found outside of the CNS (brain and spinal cord) that transmit sensory information to the CNS and allow motor commands. The motor (efferent) division of the PNS includes autonomic neurons (sympathetic, parasympathetic, and enteric neurons) which innervate involuntary smooth muscles, cardiac muscles and glands to unconsciously regulate the activity of internal organs in response to internal and external stimuli, and all the axons of the CNS motor neurons that are involved in the transmission of motor outputs. The sensory (afferent) division of the PNS includes two major categories of neurons, somatosensory (SSN) and visceral sensory (VSN) neurons, organized into cranial and spinal ganglia located along the brainstem and the spinal cord, as well as sensory neurons of the ENS. SSN respond to changes at the surface or inside of the body. They are involved in the detection and relay of sensations that organism “feels,” such as joint position, muscle stretch, touch, pressure, temperature, itch, and pain. VSN are sensors that regulate viscera physiology by sampling the internal environment, and are also involved in transmitting gustative information from the taste buds.

The important progresses made over the last 5 years in the development of sequencing technologies have evidenced an unprecedented diversity of functional sensory neuronal types in the developing and mature PNS. How this cellular diversity is created from multipotent stem/progenitor cells during embryonic development is a fundamental question in neurodevelopmental biology that remains incompletely understood. Cell fate decisions and the control of their proliferation and differentiation are well known to be controlled by environmental cues acting on intrinsic transcriptional programs. Under the influence of external cues, multipotent progenitors progressively lose their competence and acquire upon activation of specific TF networks a more differentiated state. Researches in the past decades have identified a series of TFs controlling the genesis and diversification of peripheral sensory neurons but most of the studies have focused on their role in trunk DRG. Many of these TF are, however, also expressed in other PNS sensory ganglia (Supplementary Table S1) where their functional relevance has been poorly described. In this review, we first summarize the anatomy, function and neuronal diversity of these different sensory ganglia revealed in recent studies using scRNA-seq technologies. We then provide an overview of our current knowledge of some of the main TF controlling neurogenesis and neuronal diversification, highlighting their common or divergent roles and mechanisms of action in the different sensory ganglia.

## Anatomy, Functions and Neuronal Diversity of Sensory Ganglia

In the head region of vertebrates, the PNS is organized into 12 pairs of cranial nerves, numbered I–XII. These cranial nerves contain either only motor efferent fibers [cranial nerves III (oculomotor), IV (trochlear), VI (abducens), and XII (hypoglossal)], sensory afferent fibers [cranial nerves I (olfactory), II (optic) and VIII (vestibuloacoustic)], or both fiber types [cranial nerves V (trigeminal), VII (facial), IX (glossopharyngeal), X (vagus) and XI (spinal accessory)]. As a general rule, the neuronal cell bodies of the motor division of these nerves are located within nuclei of the brainstem while their sensory division arises from dedicated head sensory ganglia. In the body region, the PNS is organized into 31 pairs of spinal nerves composed of mixed sensory and motor fibers originating from DRG and CNS motor neurons, respectively (Guthrie, 2007; Espinosa-Medina et al., 2016; Yoo and Mazmanian, 2017; Romano et al., 2019; Sudiwala and Knox, 2019).

**FIGURE 1:**
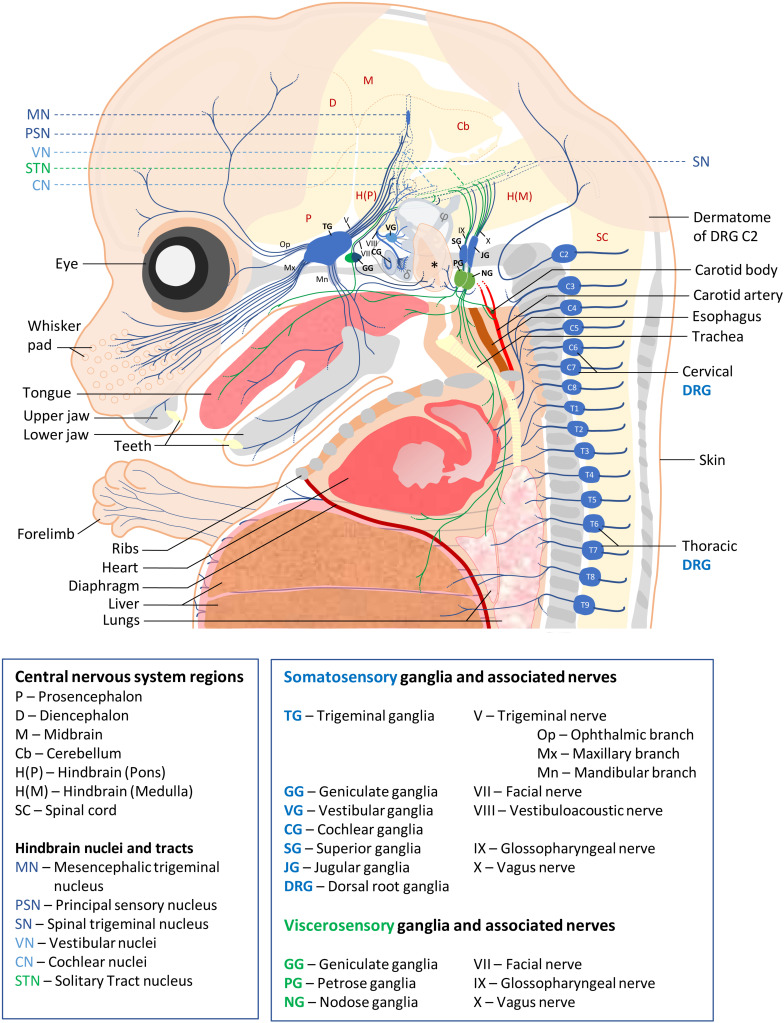
Schematic of sensory ganglia and associated nerves in a E15.5 mouse embryo. Somatosensory and visceral sensory ganglia, their peripheral innervation and targets in the CNS are represented in blue and green, respectively. The different organs and tissues are indicated. Bones are shown in gray. The external ear is indicated by an asterisk, semi-circular ducts by φ and cochlea by a δ symbol. For clarity, the olfactory epithelium, the accessory ganglia and (para-)sympathetic ganglia, the enteric nervous system and the motor efferents of cranial and spinal nerves have not been represented. Abbreviations used for all ganglia, nerves and CNS regions are indicated below the scheme.

Somatosensory neurons innervating the neck and trunk are located in DRG found in a metameric pattern on each side of the spinal cord. Those innervating the head region are located in the trigeminal ganglia, geniculate ganglia, cochlear ganglia, vestibular ganglia, superior ganglia, jugular ganglia and accessory ganglia located along the brainstem. SSN are typically pseudo-unipolar neurons with a single axon that bifurcates into two branches: a distal branch innervating target tissues and a proximal branch innervating the CNS. However, cochlear ganglia also contain some bipolar neurons while all vestibular ganglia neurons are bipolar (Carricondo and Romero-Gómez, 2019). VSN are found in the geniculate, petrose and nodose ganglia. Neurons in these ganglia are also pseudo-unipolar with fast conductive A fibers or small diameter C-fibers. The contribution of these sensory ganglia to the different cranial nerves as well as their function and neuronal diversity are summarized in Figure 1 and Table 1.

**TABLE 1:** Neuronal diversity and function of PNS sensory ganglia in adult mouse.

**Ganglia**	**Neuronal types**	**Fiber types**	**Detected and/or transmitted information**	**Identified subtypes**	**Markers expressed by mature neurons**	**Ref. to scRNA seq studies**
**TG**	**Mechanoreceptors** **C-LTMR** **Thermoceptors Pruriceptors Nociceptors**	Aβ C Aδ or C	Touch Light touch Heat and cold Irritants Noxious heat, cold, touch, chemicals	3 1 10	Piezo2, TrkB, Nav1.5 TH, Vglut3 TrpM8, Gpr26 Cd34, Piezo2, P2Y1 MrgpA3, Etv1, TrpA1, TrpV1, Tac1, CGRP, Scn10a, Grik1, MrgprD	Nguyen et al. (2017, 2019); Sharma et al. (2020)

**GG**	**Mechanoreceptors**	A	General touch	>1?	Drg11, Fxyd2, Kcns3, Brn3a, Brn3b, Piezo2	Dvoryanchikov et al. (2017); Anderson and Larson (2019); Zhang et al. (2019)
	**Chemoreceptors** (gustatory)	C	Sour Sweet Bitter? Umami? Salt? Mechano-sensitive?	[3–6]	Phox2b, P2X3, P2X2 Penk, Lypd1, Htr3a Spon1, Tac1, Itm2a Cdh13 Cdh4 Egr2 Piezo2	

**VAG**	**AG type I** **AG type II** **VG mechanoreceptors**	A A A	Sound vibration Acceleration, gravity	Type I: >3? Type II: 1 VG: N.D.	Runx1, Calb1, Ttn Lypd, Brn3a, Grm8 Trim54, Calb2, Rxrg TH, Periph, Mafb	Petitpré et al. (2018); Shrestha et al. (2018); Sun et al. (2018)

**PNG**	**Nociceptors** **Chemoreceptors** **Mechanosensors**	C C A or C	Inflammation Irritants, nutrients, Stretch, pressure	PG: N.D. NG: 18	Phox2b, P2X2, Nav1.8, Trpv1, Hoxb2-6, Tbx3, TrkC, Eya1, Nav1.1, Piezo2, P2Y1, Gpr65, Npy2r, Glp1r	Williams et al. (2016); Kupari et al. (2019); Mazzone et al. (2020)

**SJG**	**Thermoceptors** **Pruriceptors** **Nociceptors**	Aδ or C	Light touch Irritants Noxious heat, cold, touch, chemicals	SG: N.D. JG: 6	TH, Runx1, Drg11 TrpV1, TrkA, CGRP, Prdm12, TrpM8, Asic3	Kupari et al. (2019)

**DRG**	**Proprioceptor** **Mechanoreceptor** **C-LTMR** **Thermoceptors Pruriceptors Nociceptors**	Aα Aβ C Aδ or C	Limb position Touch Light touch Heat and cold Irritants Noxious heat, touch, chemical	[1–2] [2–3] 1 >5	TrkC, Parv, Runx3 TrkB, Ret, MafA Piezo2, TH, Vglut3 TrpV1, TrpM8, TrpA1, Piezo2, TH, P2X3, SST, Nav1.8, Nav1.7, TrkA, Runx1, TrpV1, TrpA1, MrgprD	Chiu et al. (2014); Usoskin et al. (2015); Li et al. (2016); Zeisel et al. (2018); Hockley et al. (2019); Sharma et al. (2020)

*TG, trigeminal ganglia; GG, geniculate ganglia; VAG, vestibuloacoustic ganglia; PNG, petrose and nodose ganglia; SJG, superior and jugular ganglia; DRG, dorsal root ganglia.*

### Trigeminal and Dorsal Root Ganglia

The trigeminal ganglia contain the sensory neurons of cranial nerve V, responsible for the detection and transmission of general somatic sensations of deep and cutaneous tissues of the head. They are therefore considered relatively similar to the DRG. Anatomically, the trigeminal nerve separates peripherally into three main branches: the ophthalmic branch innervating the supraorbital region, the maxillary branch innervating the infraorbital region and the mandibular branch innervating the lower jaw region (Haines and Mihailoff, 2018; Romano et al., 2019). DRG innervate tissues associated to local body segments (dermatomes). Cervical DRG innervate the head occipital lobe, the neck, the shoulders and forelimbs. Thoracic DRG innervate the trunk, and lumbar and sacral DRG innervate the lower body region (Haberberger et al., 2019; Noseda et al., 2019). DRG contain three major types of sensory neurons which convey specific features of somatosensation. First, proprioceptive neurons that innervate muscle spindles as well as Golgi tendon organs that sense muscle stretch and joint position, and thus give information about the general position of the body in space (proprioception). Second, low threshold mechanoreceptive (LTMR) neurons that innervate hair follicles and skin sensory structures such as Merkel cells and Meissner and Pacinian corpuscles. They respond to innocuous touch stimulations allowing the discrimination of a wide range of mechanical stimuli (vibration, pressure, stretch…). Morphologically, proprioceptors and mechanoreceptors have large soma and medium to large diameter myelinated axons (Aα and Aβ fibers, respectively). However, a subpopulation of hairy-skin innervating neurons involved in light touch stimulation and conveying affective aspects of gentle touch, called C-LTMRs, have unmyelinated axons. These neurons constitute a unique subpopulation of mechanoreceptors as they also modulate the transmission of noxious stimuli and seem to develop from precursors of the third main type of sensory neurons, the nociceptors (Lallemend and Ernfors, 2012; Delfini et al., 2013; Zimmerman et al., 2014; Emery and Ernfors, 2018; Kambrun et al., 2018; Bohic et al., 2020). This nociceptive lineage includes thermoreceptors sensing innocuous temperature variations, itch sensing pruriceptors and nociceptors sensing noxious thermal, mechanical and chemical stimuli (Emery and Ernfors, 2018). Most of the nociceptive neurons are polymodal, responding to a variety of stimuli. Nociceptors are the predominant neuronal type in trigeminal ganglia and DRG where they represent about 80% of neurons. Neurons of the nociceptive lineage are also found in superior, jugular and accessory ganglia (Cho et al., 2015; Kupari et al., 2019). Compared to proprioceptors and mechanoreceptors, nociceptive neurons are of small size and have slightly myelinated or unmyelinated axon fibers (Aδ and C, respectively). They can be further subdivided into two major subgroups, the peptidergic (PEP) and non-peptidergic (NPEP) nociceptors. The PEP nociceptors innervate the skin and deep tissues such as bones and viscera and secrete neuropeptides like Substance P or CGRP, while the NPEP nociceptors only innervate the epidermis (Yang et al., 2013; Emery and Ernfors, 2018).

The different classes of SSN in the different ganglia have also specific central termination patterns. In trigeminal ganglia, mechanoreceptors project to hindbrain neurons of the principal sensory trigeminal nucleus and of the rostral part of the SN, while trigeminal nociceptors innervate the SN *subnucleus caudalis* (Haines and Mihailoff, 2018). In DRG, each type of neuron sends stereotypical projections to specific laminae of the spinal cord. Nociceptors innervate projection neurons in laminae I and II, mechanoreceptors mainly innervate projection neurons in laminae III to V, and proprioceptors target interneurons and motor neurons in the Clarke’s column and ventral spinal cord lamina IX (Lallemend and Ernfors, 2012; Lai et al., 2016).

Molecularly, the sensitivity to specific modalities and intensities of these different types of sensory neurons is conferred by the combinatorial expression of receptors activated by defined thermal, mechanical or chemical stimuli, such as ion channels of the TRP superfamily (Patapoutian et al., 2009; Murthy et al., 2017; Bennett et al., 2019). While the molecular mechanisms of nociception have been extensively investigated since many years, molecular players underlying proprioception and touch have only recently been identified. Among them, the mechanosensitive ion channel Piezo2 appears pivotal in the detection of proprioceptive as well as innocuous and noxious mechanical stimuli (Ranade et al., 2014; Woo et al., 2015; Murthy et al., 2018). Mechanical and nociceptive information are transduced with the help of specialized cells in the periphery, with Piezo2 expressing Merkel cells being involved in mechanosensation (Ikeda et al., 2014; Ranade et al., 2014), and specialized cutaneous Schwann cells (Remak cells) forming a complex with nociceptive fibers (Abdo et al., 2019).

The molecular repertoire of DRG neurons has been first approached through next generation deep RNA-sequencing of purified neuronal populations. For example, the transcriptome of nociceptors has been evaluated using DRG neuron preparation obtained by magnetic cell sorting (Thakur et al., 2014). The molecular signature of more specific populations of nociceptors has been revealed combining genetic neuronal labeling with FACS or neuron selective chemoablation (Chiu et al., 2014; Goswami et al., 2014). The diversity of DRG neurons has been later further evaluated by analyzing the transcriptome of single cells (scRNA-seq), subsequently sorted in clusters based on their pattern of expressed genes. These studies have shown that each major category of DRG neuron can be divided in subclasses, with neurons of the nociceptive lineage showing the highest diversity, and that sensory neuron diversity varies in DRG depending on their position along the antero-posterior axis. While no final classification of DRG neurons has been established yet, these studies have provided an immense catalog of specific molecular markers that can be used to further dissect the anatomy and function of specific neuronal subtypes (Chiu et al., 2014; Usoskin et al., 2015; Li et al., 2016; Zeisel et al., 2018; Hockley et al., 2019; Sharma et al., 2020) and innervated organs (Hockley et al., 2019).

In contrast to DRG, trigeminal ganglia do not contain proprioceptive neurons. Instead, proprioceptive neurons innervating the jaw-closing muscles reside within the mesencephalic trigeminal nucleus located in the hindbrain that thus represent a unique sensory structure (Jerge, 1963; Hunter et al., 2001). Despite this major difference, a similar neuronal classification as found in DRG has been obtained in trigeminal ganglia by scRNA-seq approaches, in which groups of fast conducting mechanoreceptors, cold-sensing neurons, C-LTMR, PEP and NPEP nociceptive neurons have been described. However, while some neuronal trigeminal clusters like mechanoreceptors and itch sensing neurons closely match those described in DRG, noticeable differences in gene expression profile have been reported for other clusters (Nguyen et al., 2017, 2019; Sharma et al., 2020). In agreement with those observations, other comparative transcriptomic analyses made on dissected ganglia (Manteniotis et al., 2013), FACS sorted sensory neurons (Lopes et al., 2017) or Nav1.8 genetically labeled neurons (Megat et al., 2019) have highlighted differences between trigeminal ganglia and DRG in mouse. These studies have notably shown differences in the expression of ion channels/receptors (ex: trigeminal ganglia neurons express higher levels of ASIC1), peptides (ex: CGRP levels are higher in DRG than trigeminal ganglia) and TF (ex: Hox family members are expressed in DRG but not trigeminal ganglia neurons). For most of these markers, the functional significance of these divergences still needs to be assessed.

To date, most studies aiming at characterizing vertebrate sensory ganglia neuronal diversity and function have been undertaken using the mouse model. However, depending on the considered species, differences in sensory ganglia cellular composition and gene expression profiles have been reported, and are important to consider in translational studies. For example, the trigeminal ganglia of star-nosed mole and tactile specialist birds have a greater proportion of light touch mechanoreceptors innervating nostril appendages and beak respectively, which is considered to be an evolutionary adaptation to their lifestyle (Gerhold et al., 2013; Schneider et al., 2019). In human, the cranial embryonic nerve anatomy appears very similar to rodents (Belle et al., 2017). However, human DRG have a higher proportion of PEP nociceptors compared to rodents and have more surrounding connective tissue (Rostock et al., 2018; Haberberger et al., 2019). Comparative RNA-seq analyses of human trigeminal ganglia and lumbar DRG also reveal high similarities and analogous differences (such as Hox gene expression in DRG) as those found in mouse (Lopes et al., 2017; Ray et al., 2018).

### Geniculate Ganglia

Cranial nerve VII receives sensory fibers from the geniculate ganglia. The proximal and distal portions of the geniculate ganglia contain SSN and VSN, respectively. These SSN innervate and convey somatic (mechanical) information from the auricle and external portion of the auditory canal to the SN. Geniculate ganglia VSN essentially innervate the palate and taste buds of the anterior two thirds of the tongue and convey gustatory information to the STN located in the hindbrain (Ohman-Gault et al., 2017; Haines and Mihailoff, 2018). Three recent studies have reevaluated the diversity of geniculate ganglia neurons of adult mice, clustering the VSN into at least three groups and the SSN in only one group. While each group of VSN could potentially be linked to specific taste modalities, detailed functional and anatomical studies are needed to elucidate their peripheral target innervation and taste coding (Dvoryanchikov et al., 2017; Anderson and Larson, 2019; Zhang et al., 2019).

### Cochlear and Vestibular Ganglia

Cranial nerve VIII comprises fibers from cochlear and vestibular ganglia neurons, initially forming the vestibulo-acoustic ganglia complex during development. Cochlear ganglia, also called spiral or acoustic ganglia, contain two major types of sensory neurons conveying auditory information from the cochlea: several type I myelinated neurons innervating single cochlea inner hair cells, corresponding to ∼95% of auditory afferent fibers, and single type II unmyelinated neurons innervating multiple outer hair cells. Neurons of the vestibular ganglia transmit balance and acceleration information detected by hair cells from the inner ear semi-circular ducts, saccule and utricle (Magariños et al., 2012). Nervous fibers from the cochlear and vestibular ganglia innervate the brainstem cochlear and vestibular nuclei, respectively (Haines and Mihailoff, 2018). While the neurons of the vestibular ganglia have been poorly characterized to date, the transcriptome of the developing cochlear ganglia have been examined, revealing some TF that are uniquely expressed in cochlear ganglia and may drive auditory -specific aspects of their differentiation (Lu et al., 2011). scRNA-seq of cochlear ganglia neurons of post-natal mice support the hypothesis that the cochlear ganglia have a higher neuronal diversity than expected (Petitpré et al., 2018). In this study, mouse cochlear ganglia neurons have been classified into one group of type II neurons and at least three subtypes of type I neurons. Type I neurons subclusters are characterized by the expression of specific receptors and neurotransmitters. According to their stereotyped connection to the pillar or modiolar side of inner hair cells, they are thought to transduce specific sound information modalities based on frequencies and intensities. While the study of Petitpré et al., 2018 suggests that the different cochlear ganglia subtypes can already be distinguished at birth, two others have shown that cochlear ganglia neuron segregation requires hair cells activity dependent signaling inputs (Shrestha et al., 2018; Sun et al., 2018).

### Superior and Jugular Ganglia

The sensory fibers of cranial nerves IX and X each have two associated ganglia, the superior and jugular ganglia respectively constituting their proximal part. These ganglia contain SSN that mainly innervate ear tissues (external auditory meatus), the posterior fossa dura and tissues of the pharyngeal region, and their proximal branch makes connections with the SN. While the neuronal diversity of superior ganglia has not been characterized yet, scRNA-seq analysis has revealed that jugular ganglia neurons share a high degree of similarity with DRG neurons. Among these are cold sensing neurons expressing Trpm8, A-LTMR, C-LTMR, PEP and NPEP nociceptors. Some of them are capsaicin sensitive/TrpV1 positive and can be sensitized in inflammatory conditions (Usoskin et al., 2015; Wang et al., 2017; Kupari et al., 2019).

### Petrose and Nodose Ganglia

Petrose and nodose ganglia are the distal ganglia of cranial nerves IX and X, respectively. Petrose ganglia contain VSN that innervate structures of the respiratory system and convey taste information from the posterior third of the tongue. Nodose ganglia neurons innervate the pharyngeal area, thoracic organs and part of the digestive tract, from which they convey sensory information such as stretch and pressure and about the chemical environment (ex: inflammatory mediators). By innervating STN neurons in the brainstem, petrose and nodose ganglia neurons constitute the afferent part of reflex circuits (cardiac rhythm, peristaltism…) that are crucial for viscera activity homeostasis (Brunet and Goridis, 2008; Umans and Liberles, 2018). Until recently, despite the clinical relevance associated with vagus nerve dysfunction, only a small number of molecular markers were available to discriminate jugular ganglia SSN and nodose ganglia VSN fibers and thus their functional diversity was mostly assessed by electrophysiological recordings (Christianson et al., 2009; Ben-Menachem et al., 2015). Recent single cell transcriptomic studies of jugular and nodose ganglia have now identified several specific markers distinguishing neurons of these two ganglia. Among them, the TF Prdm12 and Phox2b, which can account for their respective somatic and visceral identities. These studies also revealed an unexpected large diversity of nodose ganglia neurons. Eighteen distinct subtypes have been defined, including stretch and volume sensing mechanoreceptors, baroreceptors, chemo- and nutrient receptors, that are dedicated to the control of the respiratory, gastrointestinal and cardiovascular systems (Wang et al., 2017; Kupari et al., 2019). By characterizing the diversity, anatomy and activity of VSN specifically innervating the lungs (Chang et al., 2015; Mazzone et al., 2020) and the digestive tract (Williams et al., 2016), a more restricted repertoire of markers has been identified in these sensory neurons that matched to some of the 18 nodose ganglia clusters mentioned above.

### Accessory Ganglia

The accessory ganglia are associated with spinal accessory cranial nerve XI whose motor fibers innervate trapezius and sternocleidomastoid muscles essential for neck and shoulder movements and laryngeal muscles required for correct movements of the vocal cords (Haines and Mihailoff, 2018). The accessory ganglia contain somatosensory nociceptive neurons that could be involved in myalgia of the trapezius and the sternocleidomastoid muscles (Bordoni et al., 2020).

## Sensory Ganglia Derive From Neurogenic Placodes and/or Neural Crest Cells

During vertebrate embryonic development, all sensory ganglia are generated from cells derived from two transient populations of embryonic multipotent stem/progenitor cells, the neural crest (NC) and/or the cranial placodes. Placodal and NC progenitors arise in close proximity to each other at the neural plate border soon after gastrulation. All placodes originate from a presumptive horseshoe shaped territory, known as the PPD, found at the anterior border of the neural plate, that subsequently resolves into discrete placodes. Neural crest cells (NCC) form along the entire border of the neural plate, except the rostral forebrain, and are positioned medially to the PPD (Saint-Jeannet and Moody, 2014). NCC are neuroectodermal cells that, after ongoing an epithelial to mesenchymal transition, delaminate from the neural tube and migrate into the embryo to give a wide range of derivatives, either directly or through the generation of pluripotent stem cell reservoirs (Boundary cap cells and Schwann cell precursors). Apart from generating neurons and glia (satellite and Schwann cells) of the PNS, NCC also give rise to autonomic and enteric neurons, melanocytes, endocrine cells, connective tissues, tendons, and the cartilage and bones that make up the cranial skeleton. NCC can be divided into distinct subpopulations based on their axial level of origin, displaying varied migratory patterns and contributing to overlapping as well as axial specific derivatives. For example, the trunk NCC generate the dorsal root and sympathetic ganglia by migrating in a segmental manner through the somites. They in contrast lack the potential to form cartilage/bone that is specific of more anterior NCC populations. Cranial placodes are thickened regions of the head ectoderm that, in addition to contributing to some cranial sensory ganglia, also give rise after more or less complex morphogenetic movements to the paired sense organs of the head (olfactory epithelium, inner ear) and other non-neurogenic specialized cells (lens, pituitary gland) (Theveneau and Mayor, 2012; Steventon et al., 2014; Furlan and Adameyko, 2018; Thiery et al., 2020).

**FIGURE 2:**
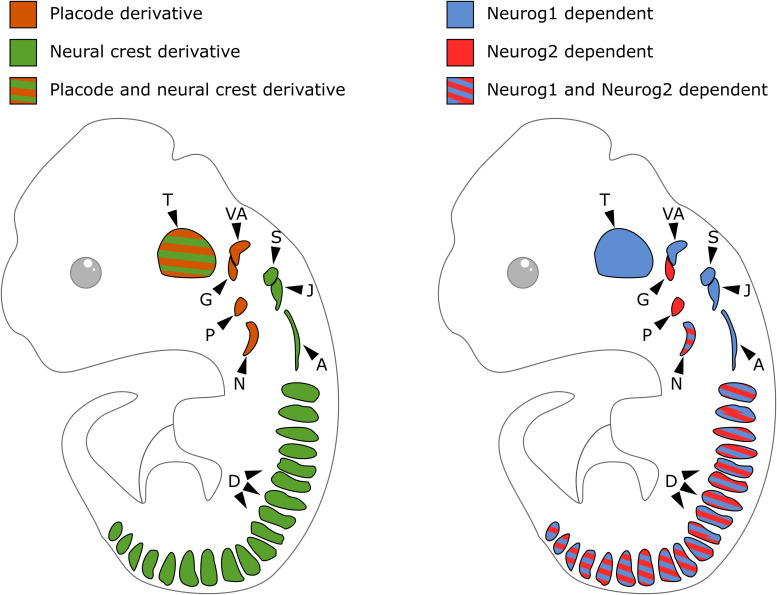
Embryonic origin and differential contribution of Neurog1 and Neurog2 to neurogenesis in the sensory ganglia. On the left, schematic of cranial sensory ganglia with indication of the contribution of neural crest (green) or placode (orange) to their formation. On the right, schematic of sensory ganglia and their developmental dependence to Neurog1 (blue) or Neurog2 (red). A, accessory ganglia; D, dorsal root ganglia; G, geniculate ganglion; J, jugular ganglion; N, nodose ganglion; P, petrose ganglion; S, superior ganglion; T, trigeminal ganglion; VA, vestibuloacoustic ganglion.

In contrast to DRG that derive only from NCC, cranial sensory ganglia have a NC or placodal origin, or both. Sensory neurons of the geniculate, petrose and nodose ganglia derive exclusively from epibranchial placodes. Those of the cochlear and vestibular ganglia derive from the otic placodes with a possible minor contribution of NCC (Breuskin et al., 2010; Freyer et al., 2011; Steventon et al., 2014). The trigeminal ganglia are of mixed origin with their proximal and distal regions respectively arising from NCC and trigeminal placodes. The superior, jugular and accessory ganglia exclusively derive from the NC (Figure 2, left). Cranial ganglia development results from the aggregation of these neurogenic placode precursors and NCC into ganglia, after delamination and migration from initial positions. In neurogenic placodes, neurogenesis occurs before the delamination step, while in the case of NCC it only starts once migrating precursors aggregate into ganglionic structures. During head morphogenesis, NCC and placodes mutually interact and these interactions drive the coordinated morphogenesis that is required for the formation of functional cranial sensory ganglia (Steventon et al., 2014; Sudiwala and Knox, 2019).

Specification of placodal and NC progenitors, their delamination, migration and diversification are well known to be controlled by different inductive signals patterning the embryonic ectoderm. These local signals set up in progenitors the expression of networks of genes encoding TF that endow them with specific mobility properties and ability to initiate a specific differentiation program. During NC formation, Wnts, FGFs and BMPs induce in the ectoderm of the gastrula embryo the expression of neural border specifier genes (i.e., Msx Pax3/7 and Dlx5/6) which define the neural plate border territory, a region primed to form NC and placodal cells. In turn, these genes in combination with signaling molecules activate “neural crest specifier” genes (like Snail/Slug, Foxd3, Twist, Id, Myc and SoxE TF) and placodal specifiers (like Six TF, in partnership with cofactors such as Eya1) that drive their specification. As the embryo acquires anterior-posterior identity, the NC becomes regionalized and cells acquire distinct developmental potential according to their axial level. Similarly, the PPD is subdivided into specific placodes with distinct developmental programs. It is now clear that an intricate array of TF controls in a stepwise process their development and diversification, leading to the differentiation of highly specialized cells. Some of these TF are known to play key roles in cell fate decision. For example, mesenchymal potential is conferred to head NCC by the expression of a single gene, Twist, and the subsequent decision of NCC to participate to the autonomic lineage requires the activation of *Phox2b* (Soldatov et al., 2019). In this review, we will focus on the transcriptional control of neurogenesis and neuronal diversification in sensory ganglia. TF controlling early PPD and NC specification and their delamination/migration, as well as those regulating the differentiation into other lineages (glial, melanocytes, mesenchymal, autonomic) are out of the scope of this review. These aspects have been extensively reviewed elsewhere (Jessen and Mirsky, 2005, 2019; Grocott et al., 2012; Lassiter et al., 2014; Saint-Jeannet and Moody, 2014; Jacob, 2015; Mort et al., 2015; Newbern, 2015; Simões-Costa and Bronner, 2015).

## Transcriptional Control of Neurogenesis and Neuronal Diversification in Sensory Ganglia

**FIGURE 3:**
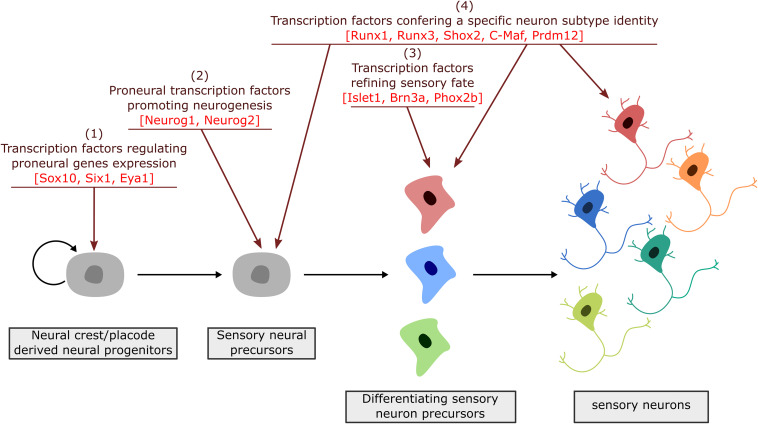
Successive steps of sensory neuron differentiation. The emergence of sensory neurons from placodal/neural crest derived cells involves transcription factors acting in a timely appropriate manner to drive sensory neuron specification and diversification. Placodal/neural crest specific TF are first required to initiate the induction of proneural factors (1). Proneural factors then act in sensory neuron precursors (neuroblasts) to select a neuronal fate and block their proliferation (2). Their activation is followed by the expression of broadly expressed TF that further refine and secure a sensory identity (3). Finally, TF with a more restricted expression pattern, acting all along the differentiation process, drive transcriptional programs required for the acquisition of dedicated sensory subtype phenotypes (4).

**FIGURE 4:**
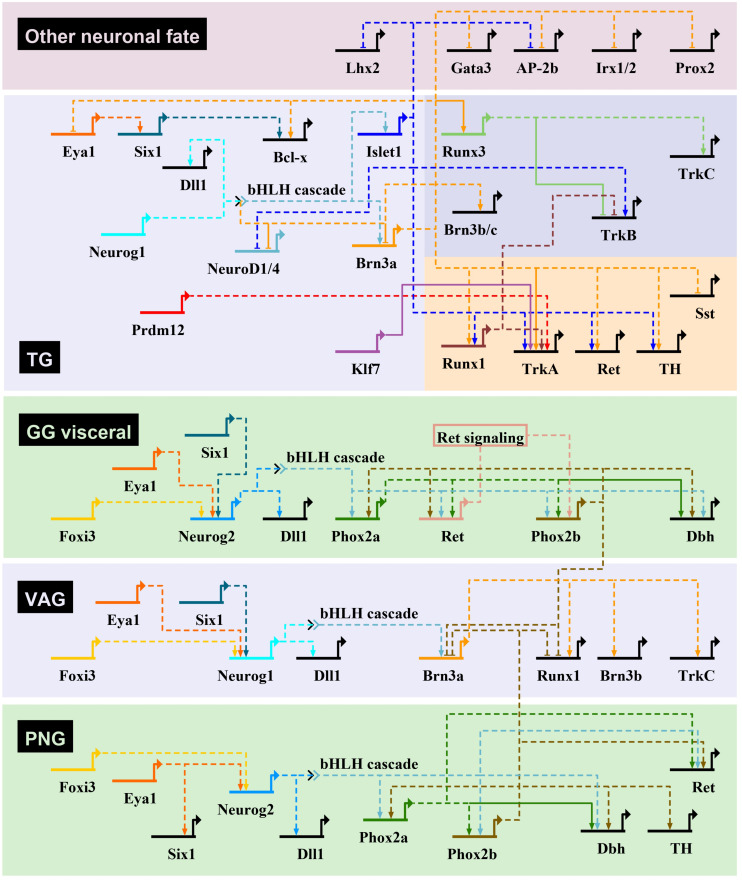
Transcriptional regulatory networks controlling neurogenesis in mouse cranial sensory ganglia. Gene regulatory interactions for the initiation of sensory neuron transcriptional programs in cranial ganglia through bHLH TF gene activation. The gene regulatory networks have been drawn using BioTapestry (Longabaugh et al., 2005), with transcriptional activation indicated by arrows, and repression by blunt-ended arrows. For the geniculate ganglia (GG), only the genes involved in VSN development are represented. In trigeminal ganglia (TG), genes specific of the mechanosensory and nociceptive lineages are shown on a darkblue and red background, respectively. Dashed lines are drawn if there is no evidence for direct regulation of target gene. The proposed gene regulatory network is based mostly from observations made in cranial ganglia from different mutant mouse lines, that have been characterized in the following references (see text for additional information): Morin et al., 1997; Pattyn et al., 1997, 1999; Fode et al., 1998; Ma et al., 1998, 2003; Huang et al., 2001; Levanon et al., 2002; Dauger et al., 2003; Trieu et al., 2003; Eng et al., 2004, 2007; Wiggins et al., 2004; Zou et al., 2004; Lei et al., 2005, 2006; Konishi et al., 2006; Lanier et al., 2007, 2009; Sun et al., 2008; Dykes et al., 2010, 2011; Senzaki et al., 2010; D’Autréaux et al., 2011; Ahmed et al., 2012; Birol et al., 2016; Donnelly et al., 2018; Bartesaghi et al., 2019; Desiderio et al., 2019. Dbh, dopamine-β-hydroxylase; Dll1, delta-like 1; PNG, petrose-nodose ganglia; Sst, somatostatin; TH, tyrosine hydroxylase; VAG, vestibuloacoustic ganglia.

During neurogenesis, NC or placode derived dividing neural progenitors give rise to neural precursors that stop to divide and progressively differentiate to form mature neurons with a specific identity, innervating specific central and peripheral targets. Downstream of the TF involved in NC and PPD specification and controlling their epithelial-mesenchymal transition and migration, a number of TF have been identified that regulate in successive steps the differentiation of peripheral sensory neurons. They can be classified into four main categories (Figure 3): (1) factors acting in undifferentiated neural progenitors that contribute to sensory neuron specification by regulating proneural gene expression, (2) proneural TF which promote the transition from dividing NC or placodal neural progenitors into post-mitotic sensory neuron precursors, (3) TF expressed broadly in differentiating post-mitotic neurons that further refine their sensory neuronal state and (4) TF assigning them a specific neuronal identity. These later TF are early and broadly co-expressed in nascent sensory neurons and acquire mutually exclusive expression patterns in differentiating subtypes (Sharma et al., 2020). In the following sections we will concentrate on the key TF that have been shown to control the differentiation and diversification of cranial sensory neurons. An outline of their reported expression is provided in Supplementary Table S1. Their known contribution to the gene regulatory network controlling the development of the different sensory ganglia in mouse is summarized in Figure 4. For some of these TF, mutations have been reported in human congenital diseases associated with sensory deficits (Table 2).

**TABLE 2:** Human disorders with sensory deficits reported in human and their associated causative genes.

**Gene name**	**Sensory deficits associated with human congenital disease**	**OMIM number and other references**
*EYA1*	Branchiootic syndrome 1 Branchiootorenal syndrome 1, with or without cataracts	602588 113650
*SIX1*	Branchiootic syndrome 3 Deafness, autosomal dominant 23	608389 605192
*NEUROG1*	Congenital cranial dysinnervation disorder (Moebius syndrome variant)	Schröder et al. (2013)
*NEUROD1*	Permanent neonatal diabetes with neurological abnormalities	Rubio-Cabezas et al. (2010)
*PHOX2B*	Central hypoventilation syndrome, congenital, with or without Hirschsprung disease	209880
*PRDM12*	Neuropathy, hereditary sensory and autonomic, type VIII (or congenital insensitivity to pain) Midface toddler excoriation syndrome (MiTES)	616488 Moss et al. (2018); Inamadar et al. (2019)
*GATA3*	Hypoparathyroidism, sensorineural deafness and renal dysplasia	146255
*C-MAF*	Ayme-Gripp syndrome	601088

### Sox10, Six and Eya Factors as Essential Upstream Regulators of Proneural Factors in NC or Placodal Derived Progenitors

*Sox10* is a member of the *Sox* gene family encoding TF with a high mobility group (HMG)- DNA-binding domain. Sox genes regulate multiple aspects of neurogenesis, including ectoderm and neuroectoderm specification and maintenance of neural stem cells (Kamachi and Kondoh, 2013). While some of them (*Sox1*, *Sox2*, and *Sox3*, constituting the *SoxB1* subgroup) are expressed in the neuroectoderm and prospective placodes, *Sox10* is expressed in migratory NC. Like other Sox factors, Sox10 is required for the maintenance of the multipotent state of NC (Kim et al., 2003). Whereas *Sox10* is turned off in the somatosensory lineage and other NC derivatives, it persists through subsequent stages of differentiation in the glial and melanocyte lineages. While Sox10 is critical for the differentiation of the glial, melanocyte and autonomic lineages, its role in DRG sensory neuron is more controversial. In mouse, DRG neuron degeneration observed in *Sox10* mutants has been interpreted as a secondary consequence of the failure of glia differentiation. Studies in zebrafish, however, suggest a direct role in sensory neuron specification by regulating the expression of the *Neurog1* proneural gene (Britsch et al., 2001; Carney et al., 2006; Delfino-Machín et al., 2017).

Besides their role in PPD formation, the homeodomain TF *Six1*, *Six2*, and *Six4* as well as their transcriptional coactivators Eya1 and Eya2 appear also to contribute to the maturation and differentiation of sensory placodes (Xu et al., 1997; Schlosser, 2014; Riddiford and Schlosser, 2016). Six1 and Eya1 are early and broadly expressed in developing cranial sensory placodes in mouse embryos. Downstream of another TF, Foxi3, necessary for priming pre-placodal ectoderm for the correct interpretation of inductive signals for the otic and epibranchial placodes (Birol et al., 2016), Six1 and Eya1 play essential roles in sensory neurogenesis that appear distinct in different ganglia. In their absence, the epibranchial placodal progenitors fail since the beginning to express the neuronal determinants Neurog2 and Phox2a which trigger neuronal differentiation in the placodal ectoderm. This failure to induce timely neuronal differentiation results in the apoptosis of the epibranchial placode progenitors (Zou et al., 2004). In contrast, in otic placodes, Eya1 and Six1 have been shown to be dispensable for the initiation of neurogenesis, but both may, however, later regulate the progressive differentiation of neuroblast precursor cells. Indeed, Eya1 and Six1 have been shown during inner ear neurogenesis to recruit the SWI/SNF chromatin-remodeling complex to mediate the transcription of *Neurog1* and *NeuroD1*. In cooperation with Sox2, Eya1 and Six1 also activate the expression of the proneural factor Atoh1 and therefore induce hair cell fate in the cochlea (Ahmed et al., 2012). Six TFs are also required for the proper development of trigeminal ganglia. In their absence, trigeminal ganglia neurons undergo extensive early apoptosis associated with reduced expression of the anti-apoptotic protein Bcl-x (Konishi et al., 2006). At the trunk level, their absence leads to the appearance of intramedullary sensory neuron-like cells, as observed in fish or amphibians. This phenotype is likely the result of altered medial NCC migration into the spinal cord and the production of immature DRG neurons and fused DRG (Yajima et al., 2014). Eya1 does not only serve as a transcriptional co-activator, but also possesses tyrosine and threonine phosphatase activities. In epibranchial placodes, via the dephosphorylation and stabilization of the Notch intracellular domain, Eya1 is also required for the generation of a non-neuronal population of cells contributing to pharyngeal arch development (Zhang et al., 2017). Thus, Eya1 and Six1 are crucial factors required for the activation or regulation in neuroblast of the neuronal developmental program of sensory ganglia. According to scRNA-seq data, Eya1 and Six1 seem to remain expressed in the adult petrose-nodose ganglionic complex but their role in differentiated VSN is unknown (Dvoryanchikov et al., 2017; Kupari et al., 2019).

### Proneural Factors Specify a Sensory Neuron Fate in Neural Precursors

Proneural factors constitute a subgroup of evolutionarily conserved basic-helix-loop-helix (bHLH) TF. In *Drosophila melanogaster*, proneural factors confer a neural identity to naïve ectodermal cells, inducing their delamination and subsequent neuronal differentiation. In contrast, in vertebrates, proneural factors are expressed in cells which have already acquired a neural identity and are sufficient to promote neurogenesis. Given their transient expression in neural progenitors, proneural factors promote neurogenesis by activating the expression of downstream target genes involved in neuronal differentiation and by inhibiting glial cell fate and cell proliferation. They are also required for the expression of the Notch ligand *Delta-like-1* to inhibit their own expression in neighboring cells via the mechanism of lateral inhibition (Fode et al., 1998; Ma et al., 1998). While in invertebrates, proneural factors are also involved in the specification of the identity of neural progenitors, the ability of their vertebrate counterparts to commit neuronal progenitors to a specific fate appears more heterogeneous. For example, in the mouse, the proneural factor Ascl1 has a higher capacity than Neurog2 to respecify the identity of neuronal populations when ectopically expressed. Ascl1 has thus properties of an instructive determinant while Neurog2 neuronal lineage specification ability relies more on the cellular context (Bertrand et al., 2002; Parras et al., 2002; Baker and Brown, 2018).

#### Neurog1 and Neurog2 in Cranial Sensory Ganglia

In the developing PNS of mouse embryos, the proneural genes *Neurog1* and *Neurog2* are transiently expressed in distinct cranial ganglia. The trigeminal, superior, jugular, accessory and vestibulo-acoustic ganglia that derive from NC and/or placodal precursors express *Neurog1*. The geniculate and petrose ganglia that derive from epibranchial placodes express *Neurog2.* The nodose ganglia that derive from placodal precursors express both *Neurog1* and *Neurog2* (Fode et al., 1998; Ma et al., 1998). While *Neurog1* remains expressed in progenitors inside condensed ganglia and in the otic cup epithelium, *Neurog2* is only detected in epibranchial placodes and migrating neuronal precursors (Figure 2, right).

The generation and analysis of *Neurog1*^–/–^ and *Neurog2*^–/–^ mutant mouse embryos have revealed their critical requirement for neuronal differentiation in cranial ganglia. In *Neurog1* mutants, trigeminal, superior, jugular, accessory and vestibulo-acoustic ganglia are lost. In *Neurog2* mutants, the delamination and differentiation of the geniculate and petrosal placode progenitors are altered, which results in delayed development of the distal portion of the geniculate ganglia and loss of the petrose ganglia. The nodose ganglion is spared in *Neurog1* or *Neurog2* single mutants but is lost when they are removed together, suggesting they have a redundant role in this ganglion (Fode et al., 1998; Ma et al., 1998; Takano-Maruyama et al., 2012; Espinosa-Medina et al., 2014). Hence, Neurog1/2 are proneural factors which are critical to initiate the neuronal differentiation of progenitors and to balance the timing of neurogenesis in cranial sensory ganglia (Bertrand et al., 2002; Huang et al., 2014).

#### Neurog1 and Neurog2 in Dorsal Root Ganglia

**FIGURE 5:**
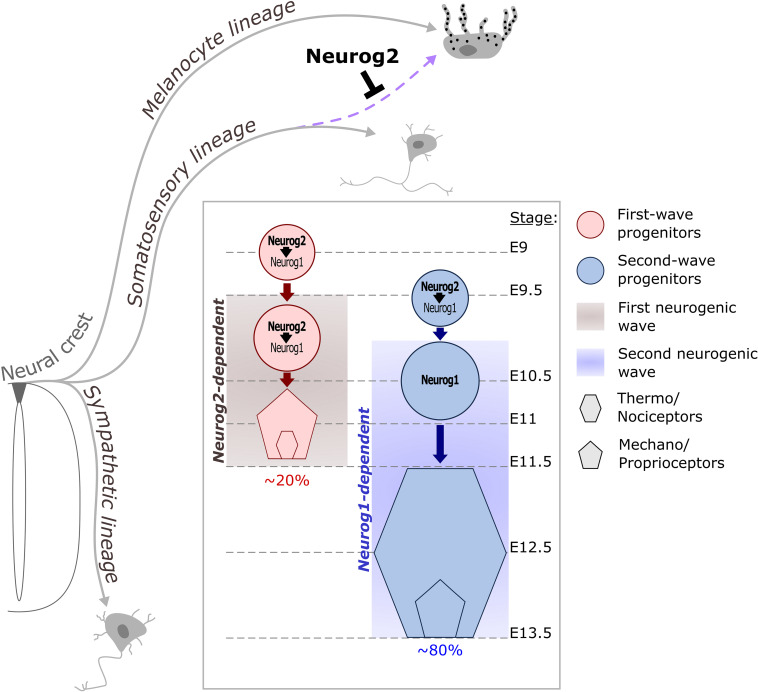
Contribution of Neurog proneural factors to DRG somatosensory neurogenesis in mouse. NCC delaminate from the dorsal part of the neural tube and differentiate into distinct lineages. Early expression of *Neurog2* in migrating NCC prevents melanocyte fate in a subpopulation of progenitors dedicated to the somatosensory lineage (purple dashed arrow). Somatosensory neurons in DRG are generated during two overlapping waves of neurogenesis. The first wave occurs between E9.5 and E11.5 and is Neurog2-dependent. This wave contributes to ∼20% of the DRG neuronal population and mostly generates mechano/proprioceptive neurons as well as a subset of nociceptors which are the large diameter nociceptors. During the second wave, Neurog2 is not sufficient to drive the differentiation of progenitors but is required to ensure the on-time onset of *Neurog1*, which depends on Neurog2 for its expression between E9.5 and E10.5. Around E11, *Neurog1* expression becomes independent of Neurog2 and can be observed until E13.5 in second wave progenitors. This second waves mainly produces small diameter nociceptor and contributes to ∼80% of the DRG neurons.

The distinct sensory neuron subtypes found in DRG are generated under the control of Neurog2 and Neurog1 in two successive neurogenic waves (Figure 5). The first wave generates most mechano/proprioceptors as well as a subpopulation of early-born myelinated nociceptors while the second wave mostly produces late-born unmyelinated neurons of the nociceptive lineage (Marmigère and Ernfors, 2007; Lallemend and Ernfors, 2012). In mouse, *Neurog2* is induced first in migrating somatosensory progenitors and remains until the end of the first neurogenic wave while *Neurog1* is expressed slightly later directly inside the DRG primordium and remains until the end of the second neurogenic wave. *Neurog1*^–^*^/^*^–^; *Neurog2*^–^*^/^*^–^ double-knockouts result in the agenesis of DRG as no sensory neuron is ever produced while *Neurog1*^–^*^/^*^–^ DRG show a specific lack of late-born nociceptive neurons. These observations have established the respective requirement of *Neurog1* and *Neurog2* for the second or first neurogenic wave (Ma et al., 1999). However, recent evidences indicate that the function of *Neurog2* is more complex than initially determined (Ventéo et al., 2019). In *Neurog2*^–^*^/^*^–^ DRG, the onset of neurogenesis from both waves is delayed as well as the induction of *Neurog1*, indicating a transient control of *Neurog2* on *Neurog1* initiation (Ma et al., 1999). The *Neurog2*^–^*^/^*^–^ neurogenesis defects are eventually compensated by *Neurog1* but result in an approximate reduction of ∼30% of all DRG neuron subtypes including those arising from the second neurogenic wave. Moreover, this delayed neurogenesis results in a brief period during which NCC putative somatosensory progenitors degenerate or adopt a melanocytic cell fate. Whether this early involvement in the bias of a NCC somatosensory identity is a specific feature of Neurog2 or more likely related to its earlier onset compared to Neurog1 remains, however, to be determined (Zirlinger et al., 2002; Soldatov et al., 2019; Ventéo et al., 2019). The specific compensatory mechanism occurring into *Neurog2*^–^*^/^*^–^ first wave progenitors has been further evidenced by the recent use of the *DBZEB;Wnt1Cre;Egr2^*DT/*+^* (DWE) mouse line in which second wave progenitors are genetically depleted without the loss of *Neurog1*, leaving only first wave progenitors (Ohayon et al., 2015). Hence, the use of a *Neurog2*^–/–^; DWE mouse line has allowed the assessment of first wave progenitors in the absence of *Neurog2* in a context where *Neurog1* can still be expressed. It has been observed that first wave progenitors have the ability to express *Neurog1* even in the absence of Neurog2, but in a delayed manner, consistent with a first phase of *Neurog1* expression dependent of Neurog2 and a later one that is not, as previously suggested by Ma et al., 1999. Interestingly, the delayed induction of *Neurog1* in *Neurog2*^–/–^; DWE mutants is coupled with a change in the proportion of neuronal subtypes compared to simple DWE mutants, with a decrease of mechano- and proprioceptors and an increase of late-born nociceptors which are normally not produced by first wave progenitors. This identity switch could be caused by a divergent specification network between Neurog1 and Neurog2. It could also represent the consequence of the observed neurogenic delay caused by the later onset of *Neurog1* and may reflect the influence of divergent environmental signals at different developmental times on the identity of sensory neuron subtypes, which still needs to be evidenced. These recent observations suggest that Neurog2 plays important functions during the first and second neurogenic waves. Two more observations further support this hypothesis: (1) Lineage analyses of Green Fluorescent Protein or Cre expressing *Neurog2* locus have revealed the labeling of the vast majority of DRG neurons, including those specific of the second wave. (2) It has been reported almost three times more supernumerary melanoblasts into the *Neurog2* mutants than in the *Neurog2*^–/–^; DWE mutants, therefore implying that around the two third of the supernumerary melanoblasts observed in the *Neurog2* mutants come from second wave NCC precursors. This suggests that *Neurog2* is expressed transiently in most, if not all sensory neuron precursors at some stage, probably before the induction of *Neurog1* (Zirlinger et al., 2002; Bartesaghi et al., 2019; Soldatov et al., 2019; Ventéo et al., 2019).

### Transcription Factors Regulating the Core Gene Expression Program of Sensory Differentiation

A number of Neurog1/2 downstream TF have been identified that are activated in differentiating post-mitotic neuronal precursors and contribute to the establishment or maintenance of the neuronal program. They belong to distinct families, including the bHLH and HD families. Among them, the bHLH TF NeuroD1, NeuroD4 and Nscl1 and the HD TF Brn3a and Islet1.

#### *NeuroD* bHLH Family Members Are Downstream Mediators of Proneural Factors

In vertebrate cranial and spinal sensory ganglia, the genes encoding the bHLH factors *NeuroD1* and *NeuroD4* are directly regulated by Neurog1 and Neurog2, and their overall expression decreases as sensory neurons mature, which makes it a good readout of differentiating neurons (Ma et al., 1996, 1998; Sommer et al., 1996; Huang et al., 2000; Bertrand et al., 2002; Ventéo et al., 2019). Despite the extensive overlap between *NeuroD1* and *Neurog1/2* expression and their structural similarities, no apparent defects in sensory ganglia have been reported into *NeuroD1*-null mice, with the exception of the vestibulo-acoustic ganglia. This could be due to the overlapping expression of *NeuroD1* and *NeuroD4* in most cranial and spinal ganglia (Miyata et al., 1999; Kim et al., 2001; Pennesi et al., 2003). NeuroD1 has also been shown to share functional redundancy with another bHLH factor, Nscl1/Nhlh1, in the developing vestibulo-acoustic and petrose ganglia (Krüger et al., 2006). Mechanistically, Neurog2 and NeuroD1 share many target genes suggesting that they may act through a common core set of TF to induce neuronal differentiation. By modulating the chromatin landscape, NeuroD1 is able to orchestrate a long-term neurogenic transcriptional program that remains active even after its expression has extinguished (Pataskar et al., 2016). Besides, bHLH TF often reciprocally regulate their expression. For example, Neurog2 induces *NeuroD1* and *NeuroD4*, which can cross-activate each other as well as other transcriptional targets (but are unable to induce *Neurog2*). These reciprocal relationships are likely to be required to robustly activate the network of other TF controlling neuronal differentiation (Fode et al., 1998; Ma et al., 1998; Seo et al., 2007).

#### Islet1 and Brn3a Are Major Regulators of Sensory Differentiation

Following the initiation of sensory neurogenesis controlled by Neurog1/2, the homeobox genes *Brn3a* (*Pou4f1*) and/or *Islet1* start to be expressed in neuron committed precursors and differentiating sensory neurons and their expression is maintained throughout life (Fedtsova and Turner, 1995). Whereas Islet1 is detected in all cranial sensory ganglia, Brn3a is restricted to SSN (D’Autréaux et al., 2011). Loss of neurons have been reported in trigeminal, geniculate, cochlear, superior, jugular ganglia and DRG of *Brn3a* mutant embryos at late stages (Eng et al., 2001; Huang et al., 2001), and in all sensory ganglia of E11.5 *Islet1* mutant embryos (Liang et al., 2011). In DRG of *Islet1/Brn3a* double knock-out embryos, sensory neurons express generic neural markers, but remain in an undifferentiated state, and fail to differentiate into functional subtypes. Transcriptomic analysis of DRG of single and double mutant embryos has revealed that they act epistatically to regulate the gene expression program of sensory differentiation (Dykes et al., 2011).

In trigeminal ganglia and DRG, Brn3a and Islet1 have a major function in the termination of the early neuronal differentiation phase by repressing *NeuroD1*, *NeuroD4*, and *Neurog1*, and in the repression of alternative genetic programs related to cardiac/cranial mesoderm and spinal cord/hindbrain development (Lanier et al., 2007, 2009; Sun et al., 2008; Dykes et al., 2011). Brn3a and Islet1 also have roles in sensory subtype specification, with proprioceptors and nociceptors having a greater dependence on Brn3a and Islet1, respectively. This has to do with Islet1 and Brn3a acting as upstream regulators of the Runt-related genes *Runx1* and *Runx3* (described below). Islet1 has been shown to be required for *Runx1* expression in trigeminal ganglia and DRG (Sun et al., 2008). Brn3a is necessary for both *Runx1* and *Runx3* in trigeminal ganglia, and may act as a direct transactivator of *Runx3* (Dykes et al., 2010; Zou et al., 2012). The failure of *Islet1* and *Brn3a* mutants to appropriately activate *Runx1/3* expression in trigeminal ganglia and DRG may account for most of their sensory phenotypes such as defective axon projections, neuron cell death and subtype specification defects. Mechanistically, Brn3a has been shown to cooperate with the pan-sensory zinc finger TF Klf7 to maintain, but not to initiate, the expression in DRG and trigeminal ganglia of the neurotrophin receptor TrkA, which plays critical roles in the survival and maturation of developing nociceptors (Huang and Reichardt, 2001; Laub et al., 2001). This appears to occur by direct binding of Brn3a and Klf7 to a *Ntrk1* enhancer that drives its expression in TrkA^+^ neurons (Ma et al., 2003; Lei et al., 2005, 2006). Brn3a also interacts with the homeodomain interacting protein kinase 2 (HIPK2) cofactor, promoting its binding to DNA but suppressing its ability to activate TrkA expression (Wiggins et al., 2004).

*Islet2*, encoding a HD TF related to Islet1, is coexpressed with *Islet1* in trigeminal, superior, jugular ganglia and DRG. However, no alteration of the development of these ganglia has been observed in *Islet2* mutant embryos, suggesting functional redundancy with Islet1 (Thaler et al., 2004). The restricted and continuous expression of *Islet2* in some adult cranial ganglia neuron clusters suggests, however, that it may have a unique function at later stages.

Like *Brn3a*, the related genes *Brn3b* (*Pou4f2*) and *Brn3c* (*Pou4f3*) are also expressed in developing somatosensory ganglia and remain expressed in some mature sensory neuron subtypes in adult mice (Badea et al., 2012). While no dramatic phenotype in sensory ganglia (and other CNS region) had been described in *Brn3b* KO and *Brn3c* KO mouse embryos (Huang et al., 2001; Zou et al., 2012; Sajgo et al., 2016), recent scRNA-seq analysis of DRG neurons of *Brn3b* and *Brn3c* mutant P0 embryos and the alterations observed in the axonal ending associated with *Brn3b* and *Brn3c* subtypes indicate that they contribute to the maturation of the specific sensory neuron subtypes in which they are expressed (Sharma et al., 2020).

### Transcription Factors Assigning a Specific Neuronal Identity

**FIGURE 6:**
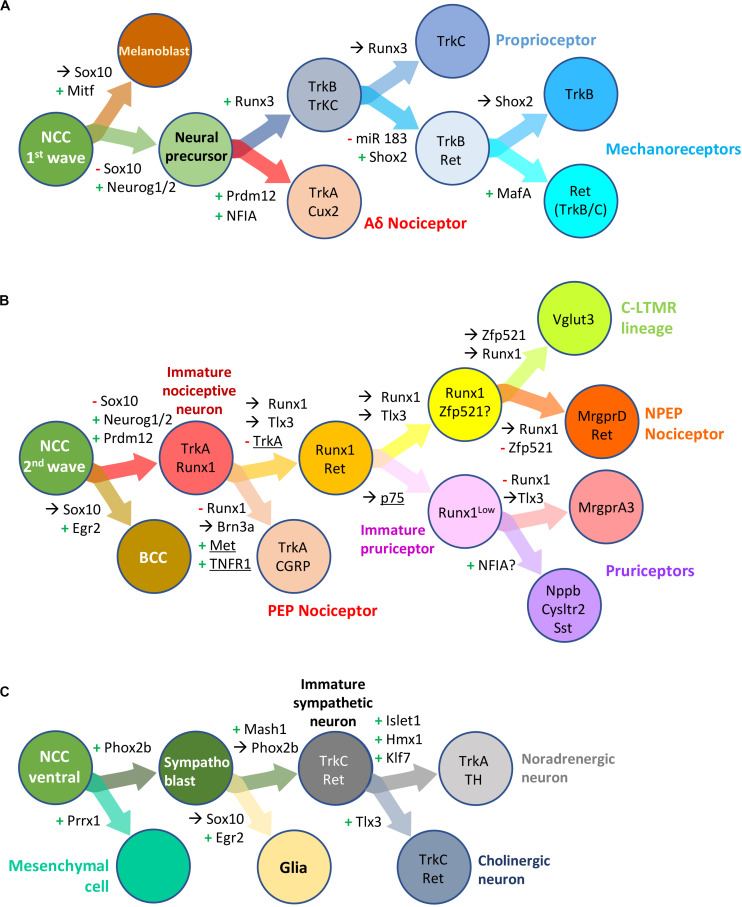
Transcription factors involved in neural crest derived dorsal root ganglia and sympathetic neuron development and diversification in mouse. Schematic overview of successive bipotent fate choices at different steps of DRG **(A,B)** and sympathetic **(C)** neuron development. **(A)** Generation of Aδ nociceptors and neurons of the mechano-/proprioceptive lineage during the first wave of DRG sensory neurogenesis. **(B)** Generation of neurons of the nociceptive lineage during the second wave of DRG sensory neurogenesis. These simplified representations highlight the influence of some transcription factor and receptors (underlined) in biasing a cell (NC derived progenitor, neural precursor or differentiating neuron) to a specific fate or neuronal lineage. +, activation; –, downregulation;→, maintained expression; NCC, neural crest cells; BCC, boundary cap cells. Note that some but not all the information in the figure has been validated using lineage tracing experiments. For more information, see the following references: Soldatov et al., 2019 (Prrx1, Neurog2); Ventéo et al., 2019 (Neurog2); Lallemend and Ernfors, 2012 (review DRG neuron specification); Peng et al., 2018 (miR 183, Shox2); Bartesaghi et al., 2019; Desiderio et al., 2019 (Prdm12, Egr2); Qi et al., 2017, 2020 (Runx1, NFIA); Lou et al., 2013, 2015 (Zfp521, Vglut3); Wheeler et al., 2014 (TNFR); Chen et al., 2017 (P75); Nagashimada et al., 2012 (Sox10, Phox2b); Furlan et al., 2013 (Hmx1, Tlx3).

NCC undergo sequential binary fate restriction decisions during their differentiation (Soldatov et al., 2019). The maturation of sensory precursors by the aforementioned pan-to-broad TF is rapidly followed or paralleled by the induction of TF with more restricted expression profiles that instruct them to adopt a specific neuronal identity. The different TF known to control binary fate decisions in DRG somatosensory neural precursors or differentiating neuron precursors are depicted in Figures 6A,B. For some of these TF, a role in the development of sympathetic ganglia has been described (Figure 6C).

#### Runx3 and the Control of the Mechano- and Proprioceptive Lineages

Members of the Runx family of TF (Runx1, Runx2, and Runx3 in mammals) are characterized by the highly conserved *RUNT* homology domain allowing nuclear translocation, DNA binding and protein-protein interactions. They also share an activation and an inhibitory domain as well as a C-terminal VWRPY motif that allow the recruitment of the Groucho/TLE co-repressors. During mouse embryonic development Runx factors have broad spatiotemporal expression patterns that overlap in some tissues. They have crucial roles in many developmental processes such as hematopoiesis, osteo-/chondrogenesis, neurogenesis, the formation of several glands and the regeneration of some tissues (Levanon et al., 2001; Wang and Stifani, 2017; Mevel et al., 2019).

In mouse DRG, *Runx3* is expressed from E10.5 in a subset of sensory precursors expressing the NT3-dependent neurotrophic receptor TrkC and becomes restricted to most developing proprioceptors from around E11.5 onward (Levanon et al., 2002; Kramer et al., 2006). Runx3 is required for the specification, early survival and proper innervation of most DRG proprioceptors partly through an indirect role in the maintenance of TrkC expression (Levanon et al., 2002; Kramer et al., 2006; Inoue et al., 2007; Lallemend et al., 2012), an activity that does not require its VWRPY motif (Kramer et al., 2006; Yarmus et al., 2006). The sustained activity of Runx3 in TrkB^+^/TrkC^+^ neurons generated during the first wave of DRG neurogenesis is also important to reduce their differentiation potential into TrkB^+^ mechanoreceptors by repressing TrkB expression either directly, or indirectly by repression of *Shox2* expression (Kramer et al., 2006; Inoue et al., 2007; Abdo et al., 2011).

Runx3 acting downstream of retinoic acid signaling appears also important in developing DRG to select which proprioceptive neurons are allowed to mature from those that will enter apoptosis, an essential selection step in the construction of functional neural circuits. Indeed, in brachial DRG, higher levels of expression of *Runx3* and TrkC were found in the developing proprioceptors that preferentially survive during the cell death period (Wang et al., 2019a). Besides, Runx3 expression level, higher at brachial and lumbar levels, controls axonal growth rate of proprioceptors and is thus critical for the development of proper central and peripheral innervation (Levanon et al., 2002; Chen A.I.et al., 2006; Lallemend et al., 2012).

After peripheral innervation, Runx3 appears necessary for the maintenance of the identity of proprioceptive sensory neurons. At this stage, NT3-TrkC signaling produced by muscle cells is required for the maintenance of its expression. Thus Runx3 acts as a terminal selector TF for DRG proprioceptive neurons (Kramer et al., 2006; Wang et al., 2019b). Other studies, however, suggest that Runx3 may be also involved in the development of a subset of DRG cutaneous mechanoreceptive neurons (Nakamura et al., 2008; Yoshikawa et al., 2013).

In mouse trigeminal ganglia, Runx3 is expressed from E10.5 and is also involved in the maintenance and amplification of TrkC expression in a subset of TrkC^+^ neurons, partly by repressing TrkB. However, compared to DRG, downregulation of TrkC expression associated with loss or downregulation of Runx3 expression is not followed by neuronal loss in trigeminal ganglia, but rather by central and peripheral innervation defects (Levanon et al., 2002; Dykes et al., 2010; Senzaki et al., 2010; Appel et al., 2016). TrkC^+^ neurons in the trigeminal ganglia are thought to correspond to mechanoreceptors mainly innervating whiskers and the skin, that are respectively dependent and independent on Runx3 (Senzaki et al., 2010). Trigeminal proprioceptors that have their cell bodies in the mesencephalic trigeminal nucleus also express TrkC but do not depend on Runx3 as assessed by the presence of jaw muscles spindles in *Runx3* KO embryos (Levanon et al., 2002).

Early Runx3 expression has also been detected in the petrose-nodose ganglionic complex (Levanon et al., 2001). Runx3 expression has been further highlighted by scRNA-seq in two VSN subtypes in nodose ganglia, both of them also characterized by TrkC expression (Kupari et al., 2019). While no important cell loss has been reported in petrose and nodose ganglia of *Runx3* KO embryos at E13.5 (Levanon et al., 2002), the function of Runx3 and its potential involvement in the regulation of TrkC expression in these ganglia awaits further investigations.

Different isoforms of Runx factors, resulting from alternative promoter expression or alternative mRNA splicing, that can additionally be subjected to several post-translational modifications, have been described *in vitro* and *in vivo*, mostly in hematopoiesis and cancer models. Indeed, each *Runx* gene can be transcribed from two promoter regions (P1 and P2) with conserved architecture. These promoters have been shown to have different activity depending on the cellular context, and mRNA transcribed from P1 or P2 would show differences in translation efficiency and stability (Mevel et al., 2019). In a recent study, it was demonstrated that *Runx3* expression in DRG and TG neurons essentially depends on its P2 promoter with differential requirement of three conserved upstream regulatory elements for distinct subtypes of TrkC neurons. Analysis of these sequences revealed potential binding sites for many TF like NeuroD, Brn3a, Islet1, Klf7 or Shox2, suggesting dynamic transcriptional integration. However, the *in vivo* relevance of these motifs has only been studied for Brn3a so far (Dykes et al., 2010; Appel et al., 2016).

#### Shox2 Is a Pivotal Factor in the Differentiation and Segregation of Touch Sensing Neurons

The TF Short stature homeobox 2 (Shox2) is initially expressed in most mouse DRG neurons from E10.5 but becomes rapidly restricted at later stages to the developing LTMR, which convey non-painful mechanical stimuli. LTMR comprise three classes of sensory mechanoreceptors that convey specific touch modalities: Aδ LTMR, Aβ rapidly adapting (RA) LTMR and Aβ slowly adapting LTMR which are also referred to as NF1, NF2 or NF3 sensory neurons based on scRNA-seq classification (Usoskin et al., 2015). These classes of neurons are discriminated by their expression level of the neurotrophic receptor TrkB which is high in NF1, low in NF2 and extinguished in NF3 that instead express TrkC. In *Shox2* KO mice, most mechanoreceptor (NF1 and NF2) TrkB^+^ neurons fail to develop concomitantly with an apparent increase of TrkC^+^ NF3 neurons. Shox2 is thus required for the development of TrkB mechanoreceptors and to repress TrkC expression (Abdo et al., 2011; Scott et al., 2011).

Some TrkB^+^ LTMR and TrkC^+^ proprioceptors arise from a common pool of TrkB^+^/TrkC^+^ precursors. Their segregation relies on Shox2 and Runx3 and their interactions. While Shox2 is required for the acquisition of a TrkB^+^ phenotype, Runx3 drives the expression of the TrkC^+^ proprioceptive fate and represses *Shox2* and *TrkB* (Abdo et al., 2011; Scott et al., 2011). Whether TrkC^+^ proprioceptive neurons are increased in *Shox2* KO mice remains unknown. Shox2 is regulated by the microRNA miR-183 cluster that represses its expression. This microRNA miR-183 cluster appears important for the timely extinction of Shox2 and thus the correct population sizes of TrkB^+^ NF1 and TrkC^+^ NF3 neurons (Peng et al., 2018).

*Shox2* is also expressed in vestibulo-acoustic, geniculate, petrose and nodose ganglia of chicken embryos (Patthey et al., 2016). In mouse, *Shox2* expression has been reported in trigeminal and geniculate ganglia. In *Shox2* KO mice, truncation of the facial nerves has been observed. However, this phenotype is likely indirect as the conditional loss of *Shox2* throughout the CNS recapitulates the nerve defects (Rosin et al., 2015). The exact function of Shox2 into cranial sensory ganglia remains thus to be defined.

#### Maf Transcription Factors Are Crucial for the Phenotypic Maturation of Mechanoreceptors

Maf (musculoaponeurotic fibrosarcoma) proteins are members of the basic-leucine-zipper (bZIP) superfamily of TF. Two members of this family have been reported so far for the development of SSN; the proto-oncogene *c-Maf* and its paralog *MafA*. *C-Maf* and *MafA* start to be detected in mouse DRG at E10.5 or E11 in post-mitotic neuronal precursors and remain expressed in DRG neurons until adulthood or post-natal stage (P15), respectively. The expression of *c-Maf* is wider than that of *MafA*, with *c-Maf* expressing neurons defining two main subgroups. A group of sensory neurons which co-expresses *c-Maf* and *MafA* together with the tyrosine-kinase receptor Ret and correspond to the RA-LTMR, and c-Maf^+^ neurons that do not express Ret or MafA and corresponds to slowly adapting mechanoreceptors, proprioceptors and a small population of nociceptors (Bourane et al., 2009; Hu et al., 2012; Wende et al., 2012). C-Maf appears to be primarily important for the development of RA-LTMR which innervate hair follicles as well as Pacinian and Meissner corpuscles of the glabrous skin. A lack of c-Maf is correlated with an aberrant morphology of Meissner corpuscles and of a large proportion of lanceolate and circumferential endings associated with hair follicles as well as a striking reduction of Pacinian corpuscles and sensory afferents innervating them. Despite this dramatic phenotype, it is interesting to note that the loss of c-Maf in DRG is not associated with any cell death but is accompanied by sensory dysfunction of the affected mechanosensory fibers which have a reduced conduction velocity and abnormal firing properties. C-Maf is required upstream of Ret and MafA for their maintenance but not their initiation (Wende et al., 2012). Noteworthily, peripheral and central projection defects observed in *c-Maf* mutants have also been reported in *Ret* mutants. This would suggest that some defects identified in *c-Maf* mutants could be caused by the loss of Ret signaling (Bourane et al., 2009; Luo et al., 2009; Wende et al., 2012). Together, these data indicate that c-Maf, albeit it does not bias sensory progenitors to the mechanoreceptive fate, is essential for their maturation.

The role played by MafA in SSN is less clear. In *MafA* mutants, the proportion of TrkB^+^ and Ret^+^ myelinated neurons are slightly affected. However, this defect is not observed in *c-Maf* mutants suggesting that this more subtle phenotype could be bypassed by the wider functions of c-Maf (Bourane et al., 2009; Hu et al., 2012).

In cranial ganglia, scRNA-seq analysis of adult murine jugular and nodose ganglia has revealed that *c-Maf* is expressed in one group of jugular ganglion neurons and in subpopulations of VSN in the nodose ganglion that have mechanosensory features (Umans and Liberles, 2018; Kupari et al., 2019). The role of *c-Maf* and *MafA* in cranial ganglia remains to be investigated.

Another recent study has identified a role for c-Maf in myelinating Schwann cells ensheathing peripheral nerves in mouse. *C-Maf* can be detected from E18.5 in these cells and is necessary to promote a sustained high level of cholesterol synthesis required for the proper maintenance of myelin sheaths (Kim et al., 2018).

#### Prdm12 Is Essential for the Specification of the Nociceptive Lineage

Prdm12 belongs to the PR-Domain containing Methyltransferase (PRDM) family of epigenetic zinc finger regulators characterized by a N-terminal PR domain that is related to the SET domain found in many histone methyltransferases. In mammals, a poly-alanine tract is found on its C-terminal part (Hohenauer and Moore, 2012; Chen et al., 2015). During *Xenopus* and mouse embryonic development, *Prdm12* is expressed in specific regions of the CNS as well as in developing trigeminal, vestibulo-acoustic, superior, jugular ganglia and DRG, where it is detected from progenitors to differentiating neurons (Kinameri et al., 2008; Thélie et al., 2015; Wang et al., 2017; Desiderio et al., 2019; Kupari et al., 2019). In trigeminal ganglia and DRG of mouse embryos, *Prdm12* expression is restricted to developing nociceptors.

The role of *Prdm12* in sensory neurogenesis has been investigated in *Xenopus* and mice. In frogs, *Prdm12* knockdown using antisense morpholinos decreases the expression of trigeminal placode neuronal markers. Conversely, its overexpression in pluripotent animal cap explants upregulates somatosensory neuronal markers (Chen et al., 2015; Matsukawa et al., 2015; Nagy et al., 2015). In mice, upon Prdm12 depletion, nociceptive precursors fail to differentiate and eventually degenerate, while mechano- and proprioceptors are unaffected. In humans carrying deleterious *PRDM12* homozygous mutations, this phenotype leads to a harmful condition termed CIP that causes a generalized inability to detect painful stimuli. Prdm12 is thus a critical determinant of nociceptive neurons (Chen et al., 2015; Bartesaghi et al., 2019; Desiderio et al., 2019).

How Prdm12 controls nociceptor development remains unclear. In mice, *Prdm12* has been shown to be required for the survival and maturation of developing nociceptors in trigeminal, superior, jugular ganglia and DRG, through its role in the initiation and maintenance of the expression of the neurotrophic receptor TrkA (Desiderio et al., 2019). As Prdm12 is already detectable in trigeminal ganglia and DRG progenitors at E9.5 while TrkA is initiated around E11.5, it may, however, play an earlier unknown TrkA independent function. Prdm12 has further been proposed to be involved in the proliferation of DRG neuronal progenitors (Bartesaghi et al., 2019). A partial loss of *Neurog1* and its downstream effectors has been reported in DRG of *Prdm12* mutants but whether these losses are a consequence of the nociceptive neuron defects or play a role in their degeneration remains unclear (Bartesaghi et al., 2019; Desiderio et al., 2019). While in *Xenopus* animal cap explants fated to the sensory lineage by overexpressing the proneural factors Neurog1 or Neurog2 as well as in human induced pluripotent stem cells differentiated into sensory neuron, Prdm12 stimulates the expression of nociceptive markers, it does not lead to a dramatic sensory neuron conversion when overexpressed in chicken NCC, suggesting that it needs an appropriate permissive environment or dedicated partners to drive nociceptor development (Bartesaghi et al., 2019; Desiderio et al., 2019). Whether Prdm12 acts during sensory neurogenesis as a repressor or an activator remains to be determined. Despite it contains a PR domain related to histone methyltransferases, Prdm12 is unable to carry such an enzymatic activity by itself. Instead, it must recruit partners such as the repressive histone methyltransferase G9a to modulate the expression of target genes (Yang and Shinkai, 2013; Thélie et al., 2015). Moreover, ChIP-seq analysis of Prdm12 binding sites have not allowed the identification of a specific putative DNA binding motif, suggesting that it does not bind DNA directly (Thélie et al., 2015). Prdm12 may thus act as a bridge to allow the recruitment of epigenetic modifiers to specific DNA-binding proteins. This mode of action would suggest that depending on the cofactors available in nociceptors, Prdm12 would act on different targets to modulate distinct timely appropriate transcriptional programs.

In adult mice, *Prdm12* remains expressed in subsets of nociceptors in trigeminal, superior, jugular ganglia and DRG, suggesting it may modulate the function of some mature nociceptors. Recently, another disorder, milder and more localized than CIP, designated MiTES has been identified in toddlers carrying biallelic expansions of the PRDM12 poly-alanine tract. These toddlers carry scratching lesions restricted to the face with no evidence of generalized pain insensitivity (Moss et al., 2018) suggesting that *Prdm12* could play non-redundant functions in cranial and spinal sensory ganglia. Whether this phenotype reflects alteration of the development or functioning of specific nociceptors remains to be investigated.

#### Runx1 and the Diversification of the Nociceptive Lineage

In mouse DRG, second wave precursors that are characterized by TrkA expression and give rise to most neurons of the nociceptive lineage start to express *Runx1* around E12.5. *Runx1* activation depends on the TF Islet1 and the epigenetic regulator Prdm12 but does not require the NGF-TrkA signaling (Kramer et al., 2006; Sun et al., 2008; Huang et al., 2015; Desiderio et al., 2019). This signaling pathway is, however, crucial to initiate the expression of its cofactor, CBF-β, that complexes with Runx1 to activate a program of gene expression (including receptors and ion channels like TrpA1, TrpM8, and MrgprD) that is specific of nociceptors (Chen C.L.et al., 2006; Huang et al., 2015).

Although Runx1 is expressed in the TrkA lineage, it is not required for the initiation of TrkA expression, and thus for nociceptor early specification and survival. Runx1 rather refines different steps of the specification of nociceptors and allows their segregation into multiple subtypes (Chen C.L.et al., 2006; Yoshikawa et al., 2007). During the maturation phase of DRG nociceptive neurons, Runx1 plays a major role in the segregation of nociceptors into PEP and NPEP subclasses, by repressing the expression of genes encoding proteins associated with the PEP transcriptional program like TrkA and CGRP, an activity that does not require its C-terminal VWRPY motif (Chen C.L.et al., 2006; Kramer et al., 2006; Yang et al., 2013). The persistent expression of Runx1 is also crucial for correct specific central and peripheral innervation of NPEP neurons (i.e., spinal cord inner lamina II and skin epidermis; Chen C.L.et al., 2006; Yoshikawa et al., 2007; Yang et al., 2013). In that segregation process, Runx1 partly cooperates with the pan-neuronal HD TF Tlx3 to promote nociceptive precursors to adopt a NPEP identity, both factors being expressed independently of each other (Lopes et al., 2012). By regulating and/or cooperating with CBF-β and other TF like Zfp521, Runx1 also participates in the diversification of VGLUT3^+^ C-LTMR, and is further required in some NPEP neurons to acquire a pruriceptor identity partly by activating *NFIA* expression (Samad et al., 2010; Lou et al., 2013, 2015; Huang et al., 2015; Qi et al., 2020). It is, however, excluded from early-born Aδ myelinated nociceptors whose specification depends on the transient activation of *NFIA* (Qi et al., 2020).

In mouse trigeminal ganglia, Runx1 is detectable around E11. Early loss of TrkA^+^ nociceptive neurons by apoptosis has been observed in trigeminal ganglia of *Runx1* KO embryos. Neuronal loss has also been observed in the vestibular portion of the *Runx1* KO vestibulo-acoustic ganglion. The underlying mechanisms have not been investigated due to embryonic lethality around E12.5 of *Runx1* KO embryos (Okuda et al., 1996; Theriault et al., 2004). *Runx1* downregulation has also been observed in trigeminal ganglia of *Brn3a* KO embryos, however, here without apparent cell loss (Dykes et al., 2010). Runx1 and Brn3a may play a role in cell survival by cooperating in the maintenance of TrkA expression. Supporting this hypothesis, *in vitro* analysis in PC12 cells have shown that Brn3a and Runx1 can activate the *Ntrk1* promoter, potentially by direct binding (Marmigère et al., 2006). The neuronal loss in *Runx1* KO trigeminal ganglia, however, rather suggests that Runx1 is required for the survival of a subset of early generated nociceptive neurons independently of NGF-TrkA signaling. Whether as in DRG, Runx1 has roles in the postnatal diversification of nociceptive neurons in trigeminal, superior and jugular ganglia has not been investigated yet.

During vestibulo-acoustic ganglia segregation, Runx1, along with other TF like Tlx3, Tbx3 and Prdm12, is preferentially expressed in the vestibular ganglion, while TF like Prox1 and Gata3 are predominantly expressed in the cochlear ganglion (Nardelli et al., 1999; Lu et al., 2011; Desiderio et al., 2019). A similar phenotype as the one observed in the vestibulo-acoustic ganglia of *Runx1* KO embryos has been described in mutants with dramatically reduced *Islet1* expression, in *Gata3* cKO mutants and in *Brn3a* KO embryos (Huang et al., 2001; Liang et al., 2011; Luo et al., 2013). However, the functions and potential interactions of these TF have not been extensively investigated. Also, cochlear or vestibular ganglia neuron loss or innervation defects observed in *Gata3* cKO and *Prox1* cKO mouse embryos may partly be caused indirectly by defects in the formation of adjacent inner ear structures, as observed when ablating Hmx2 and/or Hmx3, which are expressed in the otic vesicle but not in vestibulo-acoustic ganglia neurons (Wang et al., 2000, 2004; Fritzsch et al., 2010; Luo et al., 2013).

#### Phox2 Factors Are Essential Regulators of the Entire Visceral Reflex Circuits

The homeodomain TF Phox2a and Phox2b are unusual TF in the sense that they act as master regulators of the entire visceral reflex circuits. They control the differentiation of the afferent pathway consisting of VSN located in the geniculate, petrose and nodose ganglia and their CNS targets, neurons of the STN (Tiveron et al., 1996; Morin et al., 1997; Fode et al., 1998; Pattyn et al., 1999; Dauger et al., 2003). Phox2b is also essential for the development of the efferent pathway consisting of visceromotor neurons located in ganglia of the sympathetic, parasympathetic and ENS (Figure 6C). In *Phox2b* mutant embryos, these NC derived structures are absent or severely reduced due to impaired precursors migration and/or survival (Pattyn et al., 1999; Coppola et al., 2010b). The defective development of these structures in *Phox2b* conditional mutants may be due, at least partly, to the requirement of Phox2b to regulate the expression of the neurotrophin receptor Ret (Coppola et al., 2010a). In the CNS, *Phox2a* and *Phox2b* are required for the generation, specification and/or migration of neurons of the locus coeruleus, branchial and visceral motor neurons of the brainstem as well as oculomotor and trochlear nuclei in the isthmus region (Morin et al., 1997; Pattyn et al., 1997; Brunet and Goridis, 2008; D’Autréaux et al., 2011). Phox2a/b have also been established as regulators of the neuronal noradrenergic phenotype in the locus coeruleus as well as in sympathetic and enteric neurons where they are involved in the initiation and maintenance of the gene *Dbh* encoding for the Dopamine β-Hydroxylase, an enzyme involved in noradrenaline synthesis (Morin et al., 1997; Pattyn et al., 1999; Coppola et al., 2010a). Phox2b is further involved in the development of a population of glutamatergic neurons in the retrotrapezoid nucleus of the brainstem, which controls breathing (Dauger et al., 2003; Dubreuil et al., 2008). In line with these various functions, defective expression and/or function of *Phox2b* in mouse and human causes pathologies like CCHS, Hirschsprung disease and some types of neuroblastoma (Amiel et al., 2003; Dauger et al., 2003; Dubreuil et al., 2008; Nagashimada et al., 2012; Boeva et al., 2017). Phox2a mutations have further been linked to Congenital Fibrosis of extraocular muscles type 2 (CFEOM2), a pathological condition revealed by inherited strabismus (Nakano et al., 2001).

In cranial sensory ganglia, *Phox2a* and *Phox2b* are expressed throughout embryogenesis in geniculate, petrose and nodose ganglia and control the early steps of the VSN specification, with *Phox2a* being already expressed in epibranchial placodes and *Phox2b* being activated in aggregating neuroblasts (Pattyn et al., 1997). In the absence of either *Phox2a* or *Phox2b*, epibranchial neuroblast delamination and aggregation into ganglia seems unaffected, however, geniculate, petrose and nodose ganglia become atrophied, partly due to increased cell death (Morin et al., 1997; Fode et al., 1998; Pattyn et al., 1999; Dauger et al., 2003).

In cranial sensory ganglia as well as in oculomotor and trochlear nuclei, Phox2a controls the initiation of *Phox2b*, while in PNS autonomic components and in the hindbrain *Phox2a* expression depends on Phox2b (Morin et al., 1997; Pattyn et al., 1997, 1999). Despite Phox2a and Phox2b share an identical homeodomain and have overlapping expression patterns, they are not functionally equivalent. Indeed, constitutive ablation of *Phox2a* leads to a milder phenotype than observed in *Phox2b* KO embryos in most structures in which it is expressed downstream of Phox2b. This is the case of the sympathetic ganglia that remain relatively spared and retain a noradrenergic phenotype. They are, however, both required for the transient expression of *Dbh* that occurs during the development of geniculate, petrose and nodose ganglia (Morin et al., 1997; Pattyn et al., 1997, 1999). The specific and redundant aspects of the function of Phox2a and Phox2b have been further examined via the generation of mouse lines in which *Phox2a* has been inserted in place of *Phox2b* and *vice-versa*. While *Phox2b* can fully compensate for *Phox2a* function in cranial ganglia development, the opposite is not true as the replacement of *Phox2b* by *Phox2a* leads to embryos with smaller petrose and nodose ganglia. The molecular defects causing this late onset atrophy have, however, not been investigated (Coppola et al., 2005).

Strikingly, in the absence of Phox2b, VSN acquire a molecular signature and projection patterns akin to that of SSN. It has been suggested that Phox2b promotes a VSN over SSN identity via the repression of *Brn3a*, as reflected by their mutual exclusiveness in VSN and SSN, respectively (D’Autréaux et al., 2011). This further suggests an ontogeny mechanism involving competing programs of somatic versus visceral fate coactivated in cranial sensory precursors that would gradually switch to preferential and later exclusive expression of only one module (Soldatov et al., 2019). Determinants of SSN over VSN identity have, however, not yet been identified.

In the geniculate ganglia, *Phox2a/b-*expressing neurons also play an indirect role in the migration and aggregation of parasympathetic precursors through the facial nerve, and in the guidance of visceral motor neurons projections (Coppola et al., 2010b). The trophic support provided by geniculate ganglia neurons innervation is also required for the formation of the taste buds (Fan et al., 2019). Similarly, visceral sensory and motor fibers constituting the vagus nerve, via their guidance role in the migration of Schwann cell precursors, contribute to the development of the ENS around the esophagus and the stomach (Espinosa-Medina et al., 2017).

Two recent studies have found clues in deciphering steps of VSN diversification in geniculate ganglia, based on the dynamic expression of the TrkB and Ret receptors, and their interaction with Phox2b (Donnelly et al., 2018; Rios-Pilier and Krimm, 2019). During mouse development, until around E15.5, almost all geniculate Phox2b^+^ VSN express the BDNF receptor TrkB whose activation is important for their early survival and for tongue innervation. From that stage onward, TrkB expression becomes downregulated in about half of Phox2b^+^ neurons (Rios-Pilier and Krimm, 2019). Adult geniculate Phox2b^+^ neurons in which TrkB expression remains constant preferentially innervate Type III taste receptor cells in taste buds, while the others are thought to innervate Type II taste receptor cells and fungiform papillae epithelium (Rios-Pilier and Krimm, 2019). The GDNF receptor Ret has a biphasic function in the development and subsequent diversification of chemosensory neurons within the geniculate ganglia. It is activated by Phox2 factors early during the formation of the geniculate ganglia (Ret is transiently expressed in ∼70% of geniculate ganglia Phox2b^+^ neurons by E13.5) and in a feedback loop amplifies the expression of Phox2b during the early embryonic window before target innervation (Donnelly et al., 2018). Ret becomes extinguished perinatally and is reactivated postnatally in a subset of lingual mechanoreceptors. It is detected in ∼20% of geniculate Phox2b^+^ neurons, with only one third of geniculate Ret^+^ neurons expressing TrkB and Ret being expressed in very few (∼15%) geniculate SSN (Donnelly et al., 2018). Ret and TrkB are also widely expressed during mouse petrose and nodose ganglia development (Qian et al., 2001). Whether their expression also plays a role in neuronal diversification in these ganglia remains to be studied. *Phox2a/b* remains expressed in VSN of adult mice (Dvoryanchikov et al., 2017; Kupari et al., 2019) but their role following neurogenesis has not been characterized so far.

Phox2b is known to interact with Rnx/Tlx3, a homeodomain TF that is broadly expressed in all cranial ganglia and whose mutation, like that of *Phox2b*, also causes CCHS. In *Tlx3* KO mice pups, the respiratory failure phenotype has been attributed to developmental defects in the hindbrain, especially in the STN and in the formation of noradrenergic centers, where Tlx3 has been found to be required for the maintenance of *Phox2b* (Shirasawa et al., 2000; Qian et al., 2001; Kondo et al., 2008). *Phox2b* expression appears, however, unaffected in petrose and nodose ganglia of *Tlx3* mutant embryos (Qian et al., 2001). This could be due, however, to functional redundancy with other Tlx factors. As Tlx3 plays a role in the segregation of cholinergic sympathetic and NPEP DRG neurons (Lopes et al., 2012; Furlan et al., 2013), one cannot rule out a similar role in petrose and nodose ganglia subtype specification which could contribute to the CCHS phenotype. Several other TF have been identified, including some poorly characterized ones such as Prox2 (Nishijima and Ohtoshi, 2006), that are expressed in different types of neurons of the geniculate and nodose ganglia (Dvoryanchikov et al., 2017; Kupari et al., 2019) whose function in VSN diversification remains to be investigated.

## Discussion

In the last years, the important progresses achieved in the development of sequencing technologies have allowed the discovery of an unprecedented diversity of neurons in vertebrate CNS and PNS. Much work remains, however, to decipher the molecular mechanisms behind this diversification. To date, the involvement of several TF in the genesis and diversification of peripheral sensory neurons has been mostly studied in DRG. Many of these TF are, however, also expressed in other cranial ganglia, in which their function has mostly been poorly described. Some of them appears to have distinct function in distinct ganglia. For example, Brn3a does not seems to regulate identical targets in trigeminal, vestibulo-acoustic ganglia and DRG (Eng et al., 2004, 2007; Sherrill et al., 2019), even though a similar combination of factors governs the initial steps of neurogenesis in these ganglia. The TF Hmx1 which is expressed in all somatosensory ganglia appears to be primarily required for the development of the geniculate ganglia only (Quina et al., 2012). Those differences are most probably explained by the presence of context and time specific interaction partners and divergent chromatin landscapes, which in this case may be influenced by the cellular origin (placode versus neural crest) of the neural progenitors. Using direct neuronal programming of embryonic stem cells, it has been recently found that the two main vertebrate proneural factors, Ascl1 and Neurog2, induce different neuronal fates by binding to largely different sets of genomic sites, determined by the intrinsic activity of their bHLH domains. Because of this initial divergent binding, distinct chromatin landscapes are induced that shape the binding and function of their shared downstream TF factors during neuronal subtype specification. Thus, the regulatory activity of TF widely expressed during neuronal differentiation will not be identical when expressed downstream of Ascl1 or Neurog2 (Aydin et al., 2019).

Many TF that have an early role in sensory neuron development remain expressed until late steps of maturation or even until adulthood, suggesting they may have distinct stage dependent functions. As aforementioned, this is clearly the case for Islet1 and Runx1 in DRG nociceptive neurons (Sun et al., 2008; Samad et al., 2010; Wang et al., 2011; Qi et al., 2017). By scRNA-seq analysis of mouse DRG neurons performed at critical developmental time points, several TF have been identified as restricted to specific neuron subtypes in postmitotic differentiating cells that constitute new potential actors of their maturation. A model has been proposed in which multiple environmental cues act on developing axons of unspecified sensory neurons. Depending on the timing and trajectories of their projection patterns, these cues resolve the TF expression patterns of newborn somatosensory neurons from a coexpressed state to a subtype-restricted state, which allows their molecular, morphological and electrophysiological specialization (Faure et al., 2020; Sharma et al., 2020). How this switch occurs during cell fate decisions in neural crest remains unknown.

Till which extent some of the presented TF, acting alone or in combination, can shape the chromatin landscape associated with a specific neuronal type, and how a given cellular context dictates TF transcriptional targets remain largely unresolved questions. The rapidly growing field of multi-omic technologies, that already allows the parallel acquisition of transcriptomic data with (epi-)genomic or proteomic information from one single cell (Chappell et al., 2018; Hu et al., 2018), will probably highly contribute to our understanding of the mechanisms of cell fate acquisition during embryonic development.

## Author Contributions

SV and SD wrote the original draft of the manuscript and prepared the figures and tables. SV, SD, and EB edited and revised the text. All authors contributed to the article and approved the submitted version.

## Conflict of Interest

The authors declare that the research was conducted in the absence of any commercial or financial relationships that could be construed as a potential conflict of interest.

## Abbreviations

BDNF: brain derived neurotrophic factor
bHLH: basic helix-loop-helix
CCHS: congenital central hypoventilation syndrome
CGRP: calcitonin gene related peptide
ChIP-seq: chromatin immunoprecipitation assay followed by DNA sequencing
CIP: congenital insensitivity to pain
(RA)-LTMR: (rapidly adapting)-low-threshold mechanoreceptors
CNS: central nervous system
Dbh: dopamine- β-hydroxylase
DRG: dorsal root ganglia
ENS: enteric nervous system
FACS: fluorescence-activated cell sorting
GDNF: glial derived neurotrophic factor
HD: homeodomain
MiTES: midface toddler excoriation syndrome
NC(C): neural crest (cells)
NGF: nerve growth factor
NPEP: non-peptidergic nociceptor
PEP: peptidergic nociceptor
PNS: peripheral nervous system
PPD: pre-placodal domain
scRNA-seq: single-cell RNA sequencing
SN: spinal trigeminal nucleus
SSN: somatosensory neurons
STN: solitary tract nucleus
TF: transcription factor
TRP: transient receptor potential
VSN: visceral sensory neurons.
