# Supplementary material for: Vertebrate Sensory Ganglia: Common and Divergent Features of the Transcriptional Programs Generating Their Functional Specialization
Source: Front Cell Dev Biol. 2020 Oct 26;8:587699. doi: 10.3389/fcell.2020.587699 (PMC7649826; doi:10.3389/fcell.2020.587699)
Supplement: Supplementary file 1 [file Presentation_1.PPTX]

## Slide 1
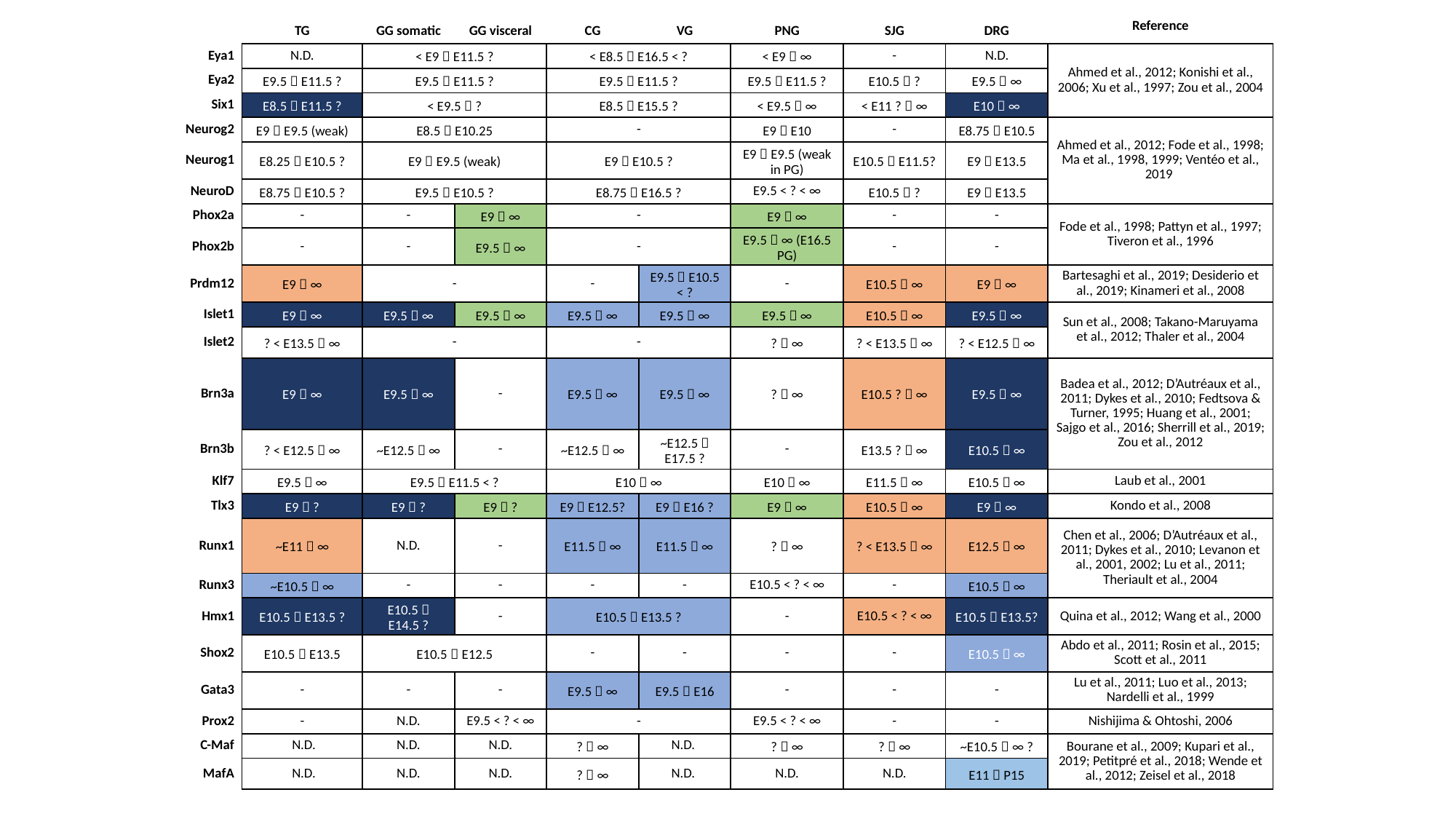

| | TG | GG somatic | GG visceral | CG | VG | PNG | SJG | DRG | Reference |
| --- | --- | --- | --- | --- | --- | --- | --- | --- | --- |
| Eya1 | N.D. | < E9  E11.5 ? | | < E8.5  E16.5 < ? | | < E9  ∞ | - | N.D. | Ahmed et al., 2012; Konishi et al., 2006; Xu et al., 1997; Zou et al., 2004 |
| Eya2 | E9.5  E11.5 ? | E9.5  E11.5 ? | | E9.5  E11.5 ? | | E9.5  E11.5 ? | E10.5  ? | E9.5  ∞ | |
| Six1 | E8.5  E11.5 ? | < E9.5  ? | | E8.5  E15.5 ? | | < E9.5  ∞ | < E11 ?  ∞ | E10  ∞ | |
| Neurog2 | E9  E9.5 (weak) | E8.5  E10.25 | | - | | E9  E10 | - | E8.75  E10.5 | Ahmed et al., 2012; Fode et al., 1998; Ma et al., 1998, 1999; Ventéo et al., 2019 |
| Neurog1 | E8.25  E10.5 ? | E9  E9.5 (weak) | | E9  E10.5 ? | | E9  E9.5 (weak in PG) | E10.5  E11.5? | E9  E13.5 | |
| NeuroD | E8.75  E10.5 ? | E9.5  E10.5 ? | | E8.75  E16.5 ? | | E9.5 < ? < ∞ | E10.5  ? | E9  E13.5 | |
| Phox2a | - | - | E9  ∞ | - | | E9  ∞ | - | - | Fode et al., 1998; Pattyn et al., 1997; Tiveron et al., 1996 |
| Phox2b | - | - | E9.5  ∞ | - | | E9.5  ∞ (E16.5 PG) | - | - | |
| Prdm12 | E9  ∞ | - | | - | E9.5  E10.5 < ? | - | E10.5  ∞ | E9  ∞ | Bartesaghi et al., 2019; Desiderio et al., 2019; Kinameri et al., 2008 |
| Islet1 | E9  ∞ | E9.5  ∞ | E9.5  ∞ | E9.5  ∞ | E9.5  ∞ | E9.5  ∞ | E10.5  ∞ | E9.5  ∞ | Sun et al., 2008; Takano-Maruyama et al., 2012; Thaler et al., 2004 |
| Islet2 | ? < E13.5  ∞ | - | | - | | ?  ∞ | ? < E13.5  ∞ | ? < E12.5  ∞ | |
| Brn3a | E9  ∞ | E9.5  ∞ | - | E9.5  ∞ | E9.5  ∞ | ?  ∞ | E10.5 ?  ∞ | E9.5  ∞ | Badea et al., 2012; D’Autréaux et al., 2011; Dykes et al., 2010; Fedtsova & Turner, 1995; Huang et al., 2001; Sajgo et al., 2016; Sherrill et al., 2019; Zou et al., 2012 |
| Brn3b | ? < E12.5  ∞ | ~E12.5  ∞ | - | ~E12.5  ∞ | ~E12.5  E17.5 ? | - | E13.5 ?  ∞ | E10.5  ∞ | |
| Klf7 | E9.5  ∞ | E9.5  E11.5 < ? | | E10  ∞ | | E10  ∞ | E11.5  ∞ | E10.5  ∞ | Laub et al., 2001 |
| Tlx3 | E9  ? | E9  ? | E9  ? | E9  E12.5? | E9  E16 ? | E9  ∞ | E10.5  ∞ | E9  ∞ | Kondo et al., 2008 |
| Runx1 | ~E11  ∞ | N.D. | - | E11.5  ∞ | E11.5  ∞ | ?  ∞ | ? < E13.5  ∞ | E12.5  ∞ | Chen et al., 2006; D’Autréaux et al., 2011; Dykes et al., 2010; Levanon et al., 2001, 2002; Lu et al., 2011; Theriault et al., 2004 |
| Runx3 | ~E10.5  ∞ | - | - | - | - | E10.5 < ? < ∞ | - | E10.5  ∞ | |
| Hmx1 | E10.5  E13.5 ? | E10.5  E14.5 ? | - | E10.5  E13.5 ? | | - | E10.5 < ? < ∞ | E10.5  E13.5? | Quina et al., 2012; Wang et al., 2000 |
| Shox2 | E10.5  E13.5 | E10.5  E12.5 | | - | - | - | - | E10.5  ∞ | Abdo et al., 2011; Rosin et al., 2015; Scott et al., 2011 |
| Gata3 | - | - | - | E9.5  ∞ | E9.5  E16 | - | - | - | Lu et al., 2011; Luo et al., 2013; Nardelli et al., 1999 |
| Prox2 | - | N.D. | E9.5 < ? < ∞ | - | | E9.5 < ? < ∞ | - | - | Nishijima & Ohtoshi, 2006 |
| C-Maf | N.D. | N.D. | N.D. | ?  ∞ | N.D. | ?  ∞ | ?  ∞ | ~E10.5  ∞ ? | Bourane et al., 2009; Kupari et al., 2019; Petitpré et al., 2018; Wende et al., 2012; Zeisel et al., 2018 |
| MafA | N.D. | N.D. | N.D. | ?  ∞ | N.D. | N.D. | N.D. | E11  P15 | |

## Slide 2
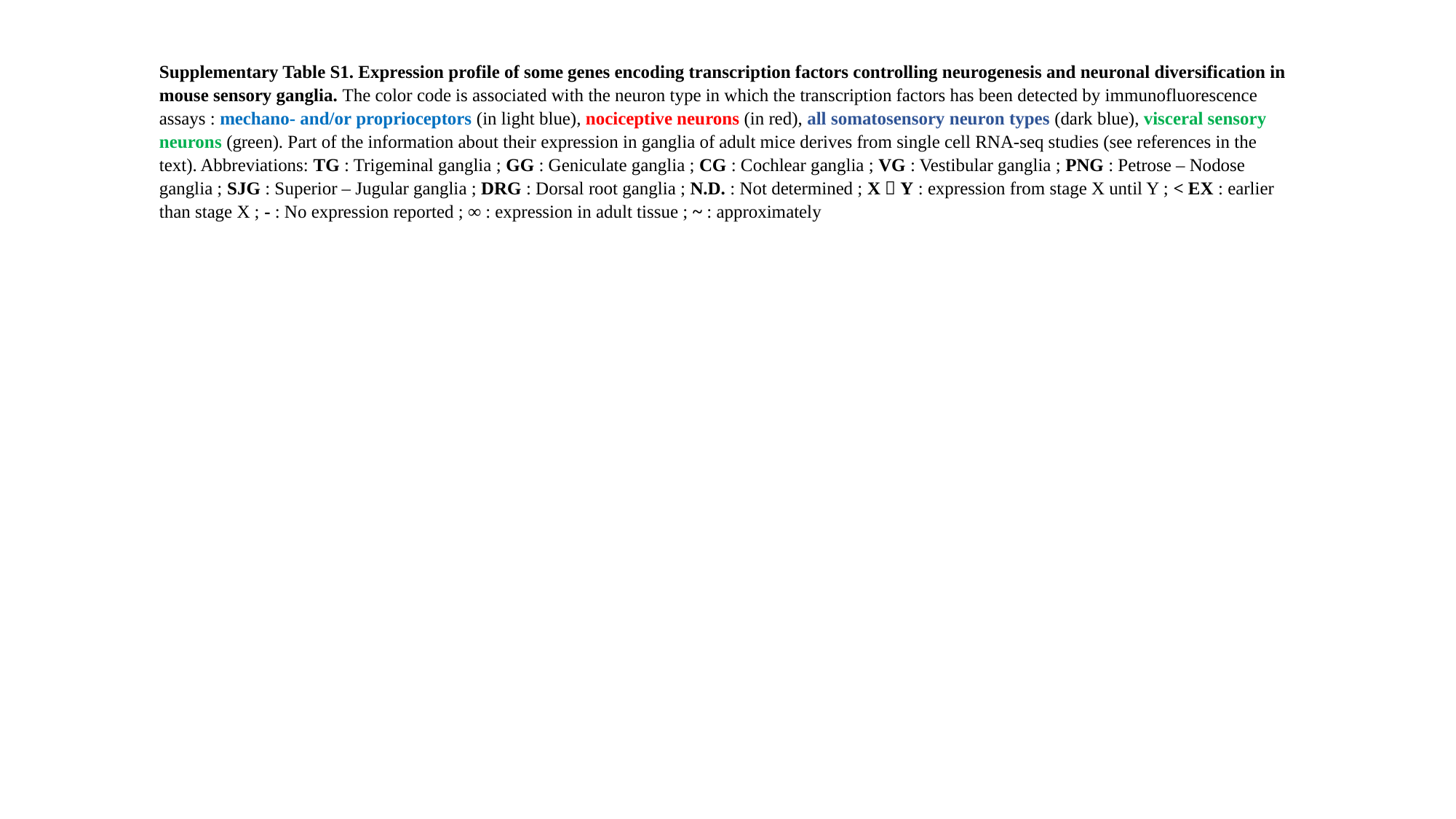

Supplementary Table S1. Expression profile of some genes encoding transcription factors controlling neurogenesis and neuronal diversification in mouse sensory ganglia. The color code is associated with the neuron type in which the transcription factors has been detected by immunofluorescence assays : mechano- and/or proprioceptors (in light blue), nociceptive neurons (in red), all somatosensory neuron types (dark blue), visceral sensory neurons (green). Part of the information about their expression in ganglia of adult mice derives from single cell RNA-seq studies (see references in the text). Abbreviations: TG : Trigeminal ganglia ; GG : Geniculate ganglia ; CG : Cochlear ganglia ; VG : Vestibular ganglia ; PNG : Petrose – Nodose ganglia ; SJG : Superior – Jugular ganglia ; DRG : Dorsal root ganglia ; N.D. : Not determined ; X  Y : expression from stage X until Y ; < EX : earlier than stage X ; - : No expression reported ; ∞ : expression in adult tissue ; ~ : approximately
